# A Dynamic Metabolic Flux Analysis of Myeloid-Derived Suppressor Cells Confirms Immunosuppression-Related Metabolic Plasticity

**DOI:** 10.1038/s41598-017-10464-1

**Published:** 2017-08-29

**Authors:** Guillaume Goffaux, Iness Hammami, Mario Jolicoeur

**Affiliations:** Research Laboratory in Applied Metabolic Engineering, Department of Chemical Engineering, École Polytechnique de Montréal, Montréal, Quebéc Canada

## Abstract

Recent years have witnessed an increasing interest at understanding the role of myeloid-derived suppressor cells (MDSCs) in cancer-induced immunosuppression, with efforts to inhibit their maturation and/or their activity. We have thus modelled MDSCs central carbon metabolism and bioenergetics dynamic, calibrating the model using experimental data on *in vitro* matured mice bone marrow cells into MDSCs. The model was then used to probe the cells metabolic state and dynamics, performing a dynamic metabolic flux analysis (dMFA) study. Indeed, MDSCs maturation correlates with a high glycolytic flux contributing to up to 95% of the global ATP turnover rate, while most of the glucose-derived carbon enters the TCA cycle. Model simulations also reveal that pentose phosphate pathway and oxidative phosphorylation activities were kept at minimal levels to ensure NADPH production and anabolic precursors synthesis. Surprisingly, MDSCs immunosuppressive activity, i.e. L-arginine uptake, metabolism and endogenous synthesis, only consumes sparse quantities of energy-rich nucleotides (ATP and NADPH). Therefore, model simulations suggest that MDSCs exhibit a heterogeous metabolic profile similar to tumour cells. This behavior is probably an indirect immunosuppressive mechanism where MDSCs reduce the availability of carbon sources in the tumour periphery microenvironment, which could explain the dysfuntion and death of immune effector cells.

## Introduction

The invasiveness of conventional anti-cancer therapies appeals to the establishment of novel therapeutic strategies. Specifically, the immunotherapy approach is gaining in interest as it showed promising results in the treatment of cancer of various types^1^. One of the mechanisms of immunotherapy specifically targets the inhibition of myeloid-derived suppressor cells (MDSCs) maturation and activity. Briefly, MDSCs are a heterogenous cell population of macrophages, dendritic cells, granulocytes, to name only the most abundant cell types^2^. These cells metabolize the semi-essential amino acid L-arginine through the enzymatic activities of arginase 1 and inducible nitric oxide synthase (ARG1 and iNOS, respectively)^3^. Both the sparse availability of L-arginine and the accumulation of nitric oxide derivatives (e.g. reactive nitrogen oxide species, RNOS) trigger the suppression of specific anti-tumour immune response^4^. Despite the considerable progress achieved in the comprehension of MDSCs biology that has resulted in multiple therapeutic approaches proposed to overcome MDSCs-mediated tumor escape, the metabolic events supporting MDSCs immunosuppressive potential are still ambiguous and poorly investigated.

Previous work on the activation of dendritic cells with lipopolysaccharides (LPS, known to activate iNOS enzyme) showed that cells undergo a PI3K/Akt-dependent metabolic transition from oxidative phosphorylation to aerobic glycolysis^5^. Likewise, LPS-activated M1 macrophages display enhanced glycolytic metabolism and reduced mitochondrial activity^6^. Therefore, we concentrated our efforts to study the central carbon metabolism and bioenergetics of MDSCs, investigating their specific requirements to acquire an immunosuppressive phenotype^7, 8^. The analysis of intracelullar metabolites concentration suggested that MDSCs exhibit a glycolytic metabolism and a high TCA cycle activity providing the immunosuppressive mechanisms with energy-rich nucleotides and carbon intermediates.

Dynamic metabolic flux analysis (dMFA) have shown being useful, as a quite unique tool, to describe extracellular and intracellular nutrients and metabolites concentration as well as fluxes transient behavior in mammalian cells^9–11^. However, such approach, as well as other metabolic modelling approaches, have not been applied yet to study the immunosuppression phenomenon, to the best of our knowledge. We thus propose here to apply a modelling approach^9–11^ previously validated on Chinese Hamster Ovary (CHO) cells^9–11^, on the maturation process of bone marrow cells into MDSCs, and thus perform dMFA to unreaveal metabolic traits of MDSCs’ maturation process.

The dynamic metabolic model combines the central carbon metabolism (CCM) and cell energetics, redox states and metabolic regulation mechanisms, and includes MDSCs immunosuppressive machinery (ARG1 and iNOS enzymes) and L-arginine endogenous synthesis reactions. The model was calibrated on experimental data of extra- and intracellular metabolites generated in a previous work^8^. We are thus first presenting a descriptive model as well as evaluating its simulation capacity. Sensitive kinetic parameters and their confidence intervals were determined to identify the key kinetic parameters and biochemical reactions to be considered as potential targets for modulating MDSCs maturation and immunosuppressive activity. Then, a dMFA study was performed through model simulations for evaluating MDSCs metabolic performance.

Model simulations confirmed the hypothesis that MDSCs maturation correlates with a high glycolytic flux sustaining ATP turnover and also supporting a high TCA cycle activity, with low but not suppressed pentose phosphate pathway and oxidative phosphorylation activities generating NADPH and anabolic precursors. Interestingly, MDSCs immunosuppressive machinery reveals not being energetically costly, while the overall nutritional profile and metabolic flux studies showed that MDSCs mimic tumour cells’ metabolic plasticity^12, 13^.

## Materials and Methods

### Cell Culture

Experimental data used herein to verify the accuracy of the metabolic model were taken from our previous work^8^ where experimental settings are well detailed.

Briefly, BM cells were extracted from 6- to 8- weeks old C57BL/6 mice (Charles River, Quebec, Canada). Animal experimentations were performed in accordance with the Canadian Council on Animal Care guidelines and the protocol was approved by the Université de Montréal’s ethical committee. Mice were kept under specific pathogen–free conditions prior to euthanasia by CO_2_.

BM cells were cultured in RPMI1640 medium (Sigma, Ontario, Canada) supplemented with 10% (v/v) irradiated fetal bovine serum (Cedarlane, Onatrio, Canada), 1 mM Sodium Pyruvate (Sigma), 50 µM β-Mercaptoethanol (Sigma), 100 U/mL Penicillin, 150 U/mL Streptomycin (Cedarlane) in a 5% CO_2_ and 37 °C incubator. The maturation of BM-derived MDSCs was performed by culturing BM cells for 96 hours in the supplemented medium mentioned above in the presence of 40 ng/mL of GM-CSF (granulocyte macrophage - colony stimulating factor) and 40 ng/mL IL-6 (Interleukin-6) as shown by Marigo *et al*.^14^.

It should be noted that the term “BM-derived MDSCs” is used when referring to experimental and simulation results on BM cells derived into MDSCs. When referring to literature, the more general term MDSCs is used because it is the terminology normally accepted for these cells.

### Metabolites Measurements

Every 24 hours, extracellular nutrients and metabolites in medium were measured using a dual-channel immobilized oxidase enzyme biochemistry analyzer (2700 SELECT, YSI Life Sciences, USA) and metabolites were extracted using cold methanol and sonication on ice method modified from Kimball *et al*.^8, 15^.

Nucleotides concentrations were measured using a 1290 UPLC system coupled to a 6460 triple quadruple mass spectrometer. A Symmetry C18 column (Waters, Canada) was employed for the separation. Mobile phases consisted of 10 mM ammonium acetate with 15 mM DMHA (N,N-dimethylhexylanine) at pH 7.0 and 50%/50% (v/v) acetonitrile, 20 mM NH_4_OAc at pH 7.0. Organic acids concentrations were assessed using the above-mentioned UPLC-MS/MS system with a Hypercarb column (Thermo Fisher, Ontario, Canada). Mobiles phases were 20 mM amoonium acetate at pH 7.5 and 10% (v/v) methanol in water.

### Model Development and Calibration

The construction steps while building the structure of the metabolic model presented in this study is based on previous models describing the central carbon metabolism of plant^16, 17^ and CHO cells^10, 11, 18^ where it showed satisfactory results simulating experimental data. The global procedure for model development is described as Supplementary material. Briefly, we have here developed a model integrating metabolic pathways known to be expressed in MDSCs, by applying a modelling approach thus previously validated with eukaryotic cells. Basically, the network of biochemical reactions was first built including central carbon metabolism as well as biochemical reactions known to be of major importance in MDSCs. Metabolic flux kinetics and regulation mechanisms were then described, based on literature on MDSCs when available, or on previous works on animal cells. The model was then describing known metabolic network in MDSCs as well as it was anchored specifically onto MDSCs by performing model structure calibration and parameters value identification thanks to experimental *in vitro* metabolomic data with these cells.

**Figure 1:**
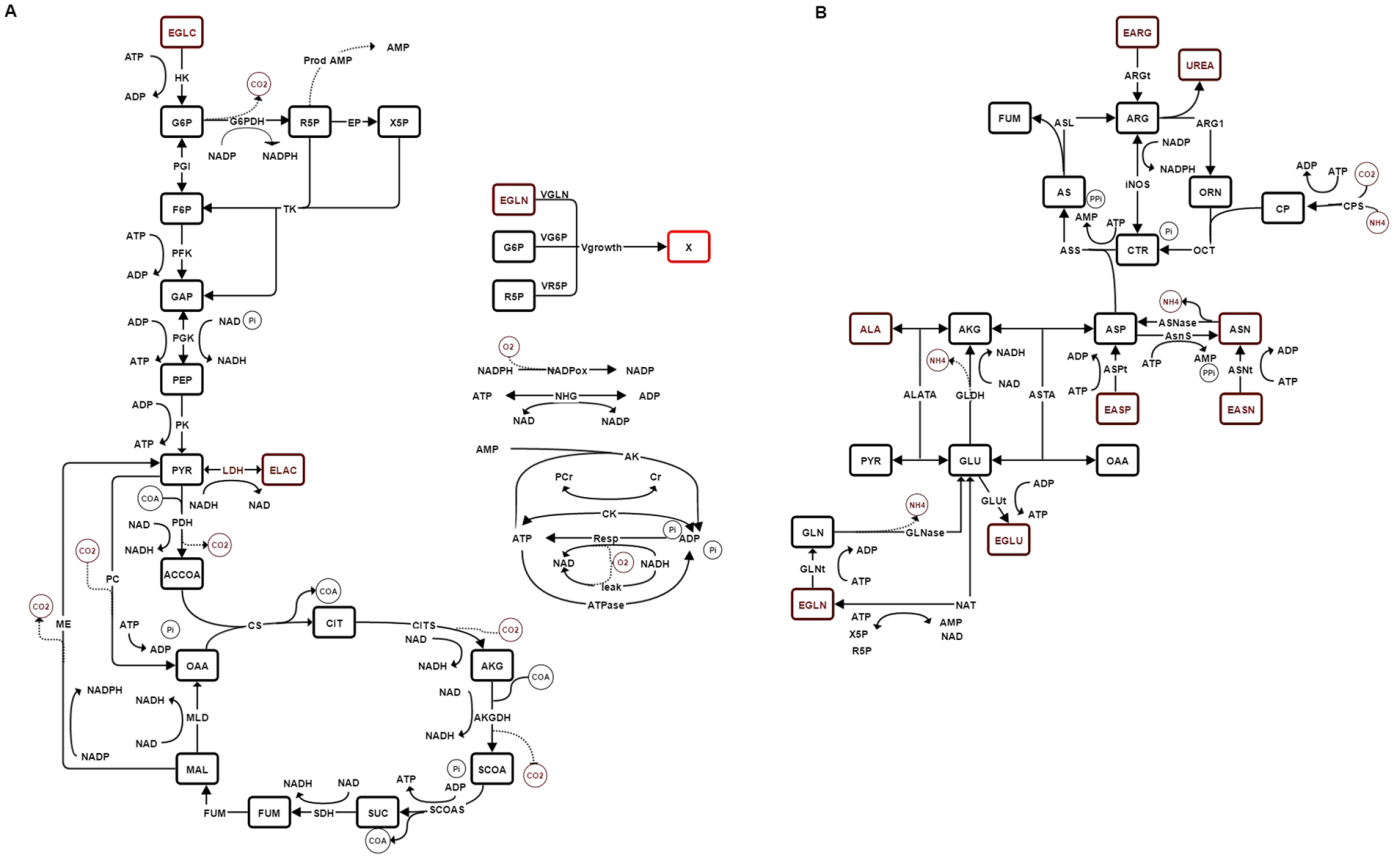
Description of the metabolic network of the model. (**A**) Central carbon metabolism and bioenergetics. (**B**) Urea cycle and amino acid catabolism. Brown: extracellular, black: intracellular. Enzymes are indicated in all reactions. All reactions are explicitely described in Table 1, including the stoichiometry.

The model metabolic network (Fig. 1) (see nomenclature in Table S1) represents the main biochemical pathways in mammalian cells including glycolysis, glutaminolysis, pentose phosphate pathway, TCA cycle, cell energetics and oxidative phosphorylation (i.e. respiration) (Fig. 1A). In addition, the urea cycle (Fig. 1B) was added in order to model the reactions involving the L-arginine metabolism, which is specifically active in the MDSCs immunosuppressive phenomenon^3^. Furthermore, the catabolic pathways related to the amino acids metabolism (notably alanine, asparagine and aspartate) were also considered since these are known to contribute as carbon source. Therefore, the model describes major inputs, such as major nutrients, and outputs, such as cell growth and the management of cell metabolites from the immunosuppressive phenomenon. This biosystem network is thus highly simplified but it considers the major set of interlinked biochemical reactions allowing to describe MDSCs behaviour.

Moreover, the modelling strategy is based on the following considerations/assumptions:

- Flux kinetics consider the effect of substrates as well as co-factors (i.e. energetic and redox nucleotides).
- Except for the amino acids alanine, asparagine, aspartate, glutamine and glutamate, all other extracellular metabolite concentrations are considered higher that the affinity constants of associated cell membrane transporters^19^. Therefore, intra- and extracellular metabolites that are consumed or secreted were taken as being at the same concentration across the cell membrane; a reliable assumption we have demonstrated in past works dealing with similar models^10, 11, 16–18^.

- The reactions of the adenylate kinase and ATPase enzymes are used to balance the time evolution of AMP, ADP and ATP energetic shuttles, with the synthesis of AMP being described.
- The model assumes non-constant energetic metabolites concentration: NAD^+^, NADH and NADP^+^, NADPH. NAD^+^ synthesis is thus described, from which NADP is synthesized.
- Enzymatic activity of the NADPH oxidase and the mitochondrial proton leak phenomenon are employed to balance the time evolution of NADP^+^/NADPH and NAD^+^/NADH, respectively.
- The stoichiometry of the reaction describing cell division and growth was based on average cell composition for CHO cells, since this information for MDSCs was not available^9^.

The final (i.e. after performing sensitivity analysis – model parameter values optimization cycle) dynamic model includes 44 reactions (Table 1) and mass balances (Table 2), with a total of 136 kinetic parameters (Table 2). The stoichiometric coefficients of the respective biosynthetic equations listed in Table 1 were taken from literature^20^. The fluxes are modeled by multiplicative Michaelis-Menten type equations and a factor of non-competitive inhibition is added when metabolites show inhibitory effects. Nutrients and metabolites as well as cell initial concentration (i.e. at t = 0) are presented in Table 3.

**Table 1:** Reactions of the metabolic network.

No.	Enzyme	Flux	Reaction
1	Hexokinase	V_HK_	EGLC + ATP → G6P + ADP
2	Phosphoglucose isomerase	V_PGI_	G6P ↔ F6P
3	Glucose-6-phosphate dehydrogenase	V_G6PDH_	G6P + 2 NADP^+^ → R5P + CO_2_ + 2 NADPH
4	Ribulose-5-phosphate epimerase	V_EP_	R5P → × 5 P
5	Transketolase	V_TK_	R5P + 2 × 5 P → 2 F6P + GAP
6	Phosphofructokinase	V_PFK_	F6P + ATP → 2 GAP + ADP
7	Phosphoglycerate kinase	V_PGK_	GAP + ADP + NAD^+^ + Pi → PEP + ATP + NADH
8	Pyruvate kinase	V_PK_	PEP + ADP → PYR + ATP
9	Lactate dehydrogenase	V_LDH_	PYR + NADH ↔ ELAC + NAD^+^
10	Pyruvate dehydrogenase	V_PDH_	PYR + CoA + NAD^+^ → ACCoA + CO_2_ + NADH
11	Citrate synthase	V_CS_	ACCoA + OAA → CIT + CoA
12	Aconitase/isocitrate dehydrogenase	V_CITS_	CIT + NAD^+^ → AKG + CO_2_ + NADH
13	Alpha ketoglutarate dehydrogenase	V_AKGDH_	AKG + CoA + NAD^+^ → SCoA + CO_2_ + NADH
14	Succinyl coenzyme A synthetase	V_SCOAS_	SCoA + ADP + Pi → SUC + CoA + ATP
15	Succinate dehydrogenase	V_SDH_	SUC + 2/3 NAD^+^ → FUM + 2/3 NADH
16	Fumarase	V_FUM_	FUM → MAL
17	Malate dehydrogenase	V_MLD_	MAL + NAD^+^ → OAA + NADH
18	Malic enzyme	V_ME_	MAL + NADP^+^ → PYR + CO_2_ + NADPH
19	Pyruvate carboxylase	V_PC_	PYR + CO_2_ + ATP → OAA + ADP + Pi
20	Alanine aminotranferase	V_AlaTA_	PYR + GLU → AKG + ALA
21	Glutamine transport	V_GlnT_	EGLN + ATP ↔ GLN + ADP
22	Glutamate transport	V_GluT_	GLU + ADP + Pi → EGLU + ATP
23	Glutamine synthetase	V_GLNase_	GLN ↔ → GLU + ENH_4_
24	Glutamate dehydrogenase	V_GLDH_	GLU + NAD^+^ → AKG + NADH + ENH_4_
25	Aspartate aminotransferase	V_ASTA_	GLU + OAA → AKG + ASP
26	Aspartate transporter	V_AspT_	EASP + ATP ↔ ASP + ADP
27	Asparagine transporter	V_AsnT_	EASN + ATP ↔ ASN + ADP
28	Asparagine synthetase	V_AsnS_	ASP + ATP → ASN + AMP + PPi
29	Asparaginase	V_Asnase_	ASN → ASP + ENH_4_
30	Arginine transporter	V_ArgT_	EARG + ATP → ARG + ADP
31	Argininosuccinate lyase	V_ASL_	AS → ARG + FUM
32	Arginase 1	V_Arg1_	ARG + H_2_O → ORN + Urea
33	Ornithine transcarbamylase	V_OTC_	ORN + CP → CTR + Pi
34	Nitric oxide synthase	V_iNOS_	ARG + 3/2 NADP^+^ → CTR + 3/2 NADPH
35	Argininosuccinate synthase	V_ASS_	CTR + ASP + ATP → AS + AMP + PPi
36	Carbamoyl phosphate synthetase	V_CPS_	CO_2_ + NH_4_ + 2 ATP → CP + 2 ADP + Pi
37	Nucleotide synthesis	V_PrAMP_	R5P → AMP
38	Creatine kinase	V_CK_	PCr + ADP ↔ Cr + ATP
39	NADPH oxidase	V_NADPHox_	NADPH + 2 O_2 → _NADP^+^ + 2 O_2_^−^ + H^+^
40	Proton leak	V_leak_	O_2_ + 2 NADH → 2NAD^+^ + 2 H_2_O
41	Respiration	V_Resp_	O_2_ + 2 NADH + 4 ADP + 4 Pi → 4 ATP + 2 NAD^+^ + 2 H_2_O
42	ATPase	V_ATPase_	ATP → ADP + Pi
43	Adenylate kinase	V_AK_	ATP + AMP → 2 ADP
44	NAD synthesis	V_NAT_	R5P + × 5 P + EGLN + 2 ATP ↔ GLU + NAD + AMP
45	NADP synthesis	V_NHG_	NAD + ATP ↔ NADP + ADP
46	Cell growth rate	V_growth_	α G6P + β R5P + γ EGln + δ ATP → X + δ ADP α = 3.18 × 10^−7^, β = 1.50 × 10^−8^, γ = 5.03 × 10^−9^, δ = 3.15 × 10^−8^

N.B. H^+^, O_2_, CO_2_, Pi, PPi and H_2_O were not considered in mass balances.

**Table 2:** Equations of kinetic fluxes and parameter values.

Number	Equation	Parameter values
1	${{\rm{V}}}_{\mathrm{HK}}={{\rm{V}}}_{\max ,\mathrm{HK}}\frac{\mathrm{EGLC}}{\mathrm{EGLC}+{{\rm{K}}}_{1,\mathrm{EGLC}}}\frac{\mathrm{ATP}}{\mathrm{ATP}+{{\rm{K}}}_{1,\mathrm{ATP}}}$	V_max,HK_ = 5.08 × 10^−4^ K_1,ATP_ = 1.08 × 10^−5^ K_1,EGLC_ = 10^−12^
2	${{\rm{V}}}_{\mathrm{PGI}}={{\rm{V}}}_{\max ,\mathrm{PGI}}^{{\rm{f}}}\frac{{\rm{G}}6{\rm{P}}}{{\rm{G}}6{\rm{P}}+{{\rm{K}}}_{2,{\rm{G}}6{\rm{P}}}}-{{\rm{V}}}_{\max ,\mathrm{PGI}}^{{\rm{r}}}\frac{{\rm{F}}6{\rm{P}}}{{\rm{F}}6{\rm{P}}+{{\rm{K}}}_{2,{\rm{F}}6{\rm{P}}}}$	V^f^_max,PGI_ = 3.53 × 10^−4^ V^r^_max,PGI_ = 9.47 × 10^−7^ K_2,F6P_ = 1.93 × 10^−5^ K_2,G6P_ = 1.04 × 10^−6^
3	${{\rm{V}}}_{{\rm{G}}6\mathrm{PDH}}={{\rm{V}}}_{\max ,{\rm{G}}6\mathrm{PDH}}\frac{{\rm{G}}6{\rm{P}}}{{\rm{G}}6{\rm{P}}+{{\rm{K}}}_{3,{\rm{G}}6{\rm{P}}}}\frac{\mathrm{NADP}}{\mathrm{NADP}+{{\rm{K}}}_{3,\mathrm{NADP}}}\frac{\mathrm{ATP}}{\mathrm{ATP}+{{\rm{K}}}_{3,\mathrm{ATP}}}$	V_max,G6PDH_ = 7.62 × 10^−5^ K_3,ATP_ = 1.03 × 10^−12^ K_3,G6P_ = 1.32 × 10^−6^ K_3,NADP_ = 10^−12^
4	${\text{V}}_{\text{EP}}\text{=}{\text{V}}_{\text{max,EP}}\frac{\text{R5P}}{\text{R5P+}{\text{K}}_{\text{4,R5P}}}$	V_max,EP_ = 6.74 × 10^−5^ K_4,R5P_ = 10^−6^
5	${{\rm{V}}}_{\mathrm{TK}}={{\rm{V}}}_{\max ,\mathrm{TK}}\frac{{\rm{X}}5{\rm{P}}}{{\rm{X}}5{\rm{P}}+{{\rm{K}}}_{5,{\rm{X}}5{\rm{P}}}}\frac{{\rm{R}}5{\rm{P}}}{{\rm{R}}5{\rm{P}}+{{\rm{K}}}_{5,{\rm{R}}5{\rm{P}}}}$	V_max,TK_=3.37 × 10^−5^ K_5,R5P_ = 10^−6^ K_5,X5P_ = 10^−6^
6	${{\rm{V}}}_{\mathrm{PFK}}={{\rm{V}}}_{\max ,\mathrm{PFK}}\frac{{\rm{F}}6{\rm{P}}}{{\rm{F}}6{\rm{P}}+{{\rm{K}}}_{6,{\rm{F}}6{\rm{P}}}}\frac{\mathrm{ATP}}{\mathrm{ATP}+{{\rm{K}}}_{6,\mathrm{ATP}}}$	V_max,PFK_ = 3.42 × 10^−4^ K_6,ATP_ = 1.03 × 10^−12^ K_6,F6P_ = 2.83 × 10^−7^
7	${{\rm{V}}}_{\mathrm{PGK}}={{\rm{V}}}_{\max ,\mathrm{PGK}}\frac{\mathrm{GAP}}{\mathrm{GAP}+{{\rm{K}}}_{7,\mathrm{GAP}}}\frac{\mathrm{NAD}}{\mathrm{NAD}+{{\rm{K}}}_{7,\mathrm{NAD}}}\frac{\mathrm{ADP}}{\mathrm{ADP}+{{\rm{K}}}_{7,\mathrm{ADP}}}$	V_max,PGK_ = 7.35 × 10^−3^ K_7,ADP_ = 4.75 × 10^−10^ K_7,GAP_ = 10^−6^ K_7,NAD_ = 10^−8^
8	${{\rm{V}}}_{\mathrm{PK}}={{\rm{V}}}_{\max ,\mathrm{PK}}\frac{\mathrm{PEP}}{\mathrm{PEP}+{{\rm{K}}}_{8,\mathrm{PEP}}}\frac{\mathrm{ADP}}{\mathrm{ADP}+{{\rm{K}}}_{8,\mathrm{ADP}}}$	V_max,PK_ = 9.79 × 10^−4^ K_8,ADP_ = 4.75 × 10^−10^ K_8,PEP_ = 4.20 × 10^−7^
9	${{\rm{V}}}_{\mathrm{LDH}}={{\rm{V}}}_{\max ,\mathrm{LDH}}\frac{\mathrm{PYR}}{\mathrm{PYR}+{{\rm{K}}}_{9,\mathrm{PYR}}}\frac{\mathrm{NADH}}{\mathrm{NADH}+{{\rm{K}}}_{9,\mathrm{NADH}}}$	V_max,LDH_ = 9.99 × 10^−4^ K_9,NADH_ = 9.86 × 10^−6^ K_9,PYR_ = 1.65 × 10^−12^
10	${{\rm{V}}}_{\mathrm{PDH}}={{\rm{V}}}_{\max ,\mathrm{PDH}}\frac{\mathrm{PYR}}{\mathrm{PYR}+{{\rm{K}}}_{10,\mathrm{PYR}}}\frac{\mathrm{NAD}}{\mathrm{NAD}+{{\rm{K}}}_{10,\mathrm{NAD}}}$	V_max,PDH_ = 1.87 × 10^−1^ K_10,NAD_ = 10^−8^ K_10,PYR_ = 1.48 × 10^−2^
11	${{\rm{V}}}_{\mathrm{CS}}={{\rm{V}}}_{\max ,\mathrm{CS}}\frac{\mathrm{OAA}}{\mathrm{OAA}+{{\rm{K}}}_{11,\mathrm{OAA}}}\frac{\mathrm{ACCoA}}{\mathrm{ACCoA}+{{\rm{K}}}_{11,\mathrm{ACCoA}}}$	V_max,CS_ = 5.00 × 10^−5^ K_11,ACCoA_ = 10^−^10^−4^ K_11,OAA_ = 10^−4^
12	${{\rm{V}}}_{\mathrm{CITS}}={{\rm{V}}}_{\max ,\mathrm{CITS}}\frac{\mathrm{CIT}}{\mathrm{CIT}+{{\rm{K}}}_{12,\mathrm{CIT}}}\frac{\mathrm{NAD}}{\mathrm{NAD}+{{\rm{K}}}_{12,\mathrm{NAD}}}$	V_max,CITS_ = 9.33 × 10^−6^ K_12,CIT_ = 1.22 × 10^−5^ K_12,NAD_ = 10^−8^
13	${{\rm{V}}}_{\mathrm{AKGDH}}={{\rm{V}}}_{\max ,\mathrm{AKGDH}}\frac{\mathrm{AKG}}{\mathrm{AKG}+{{\rm{K}}}_{13,\mathrm{AKG}}}\frac{\mathrm{NAD}}{\mathrm{NAD}+{{\rm{K}}}_{13,\mathrm{NAD}}}$	V_max,AKGDH_ = 1.14 × 10^−5^ K_13,AKG_ = 2.77 × 10^−7^ K_13,NAD_ = 10^−8^
14	${{\rm{V}}}_{\mathrm{SCOAS}}={{\rm{V}}}_{\max ,\mathrm{SCOAS}}\frac{\mathrm{SCOA}}{\mathrm{SCOA}+{{\rm{K}}}_{14,\mathrm{SCOA}}}\frac{\mathrm{ADP}}{\mathrm{ADP}+{{\rm{K}}}_{14,\mathrm{ADP}}}$	V_max,SCOAS_ = 2.45 × 10^−6^ K_14,ADP_ = 2.00 × 10^−7^ K_14,SCOA_ = 4.77 × 10^−5^
15	${{\rm{V}}}_{\mathrm{SDH}}={{\rm{V}}}_{\max ,\mathrm{SDH}}\frac{\mathrm{SUC}}{\mathrm{SUC}+{{\rm{K}}}_{15,\mathrm{SUC}}}\frac{\mathrm{NAD}}{\mathrm{NAD}+{{\rm{K}}}_{15,\mathrm{NAD}}}$	V_max,SDH_ = 4.59 × 10^−3^ K_15,NAD_ = 10^−8^ K_15,SUC_ = 1.27 × 10^−2^
16	${{\rm{V}}}_{\mathrm{FUM}}={{\rm{V}}}_{\max ,\mathrm{FUM}}\frac{\mathrm{FUM}}{\mathrm{FUM}+{{\rm{K}}}_{16,\mathrm{FUM}}}$	V_max,FUM_ = 5.03 × 10^−6^ K_16,FUM_ = 3.44 × 10^−6^
17	${{\rm{V}}}_{\mathrm{MLD}}={{\rm{V}}}_{\max ,\mathrm{MLD}}\frac{\mathrm{MAL}}{\mathrm{MAL}+{{\rm{K}}}_{17,\mathrm{MAL}}}\frac{\mathrm{NAD}}{\mathrm{NAD}+{{\rm{K}}}_{17,\mathrm{NAD}}}$	V_max,MLD_ = 4.70 × 10^−6^ K_17,MAL_ = 3.01 × 10^−6^ K_17,NAD_ = 10^−12^
18	${{\rm{V}}}_{\mathrm{ME}}={{\rm{V}}}_{\max ,\mathrm{ME}}\frac{\mathrm{MAL}}{\mathrm{MAL}+{{\rm{K}}}_{18,\mathrm{MAL}}}\frac{\mathrm{NADP}}{\mathrm{NADP}+{{\rm{K}}}_{18,\mathrm{NADP}}}$	V_max,ME_ = 8.01 × 10^−7^ K_18,MAL_ = 9.01 × 10^−8^ K_18,NADP_ = 10^−12^
19	${{\rm{V}}}_{\mathrm{PC}}={{\rm{V}}}_{\max ,\mathrm{PC}}\frac{\mathrm{PYR}}{\mathrm{PYR}+{{\rm{K}}}_{19,\mathrm{PYR}}}\frac{\mathrm{ATP}}{\mathrm{ATP}+{{\rm{K}}}_{19,\mathrm{ATP}}}$	V_max,PC_ = 1.23 × 10^−5^ K_19,ATP_ = 10^−12^ K_19,PYR_ = 1.03 × 10^−5^
20	${{\rm{V}}}_{\mathrm{AlaTA}}={{\rm{V}}}_{\max ,\mathrm{ALATA}}\frac{\mathrm{PYR}}{\mathrm{PYR}+{{\rm{K}}}_{20,\mathrm{PYR}}}\frac{\mathrm{GLU}}{\mathrm{GLU}+{{\rm{K}}}_{20,\mathrm{GLU}}}$	V_max,AlaTA_ = 10^−6^ K_20,GLU_ = 10^−4^ K_20,PYR_ = 10^−12^
21	${{\rm{V}}}_{\mathrm{GLNT}}={{\rm{V}}}_{\max ,\mathrm{GLNT}}\frac{\mathrm{EGLN}}{\mathrm{EGLN}+{{\rm{K}}}_{21,\mathrm{EGLN}}}\frac{\mathrm{ATP}}{\mathrm{ATP}+{{\rm{K}}}_{21,\mathrm{ATP}}}$	V_max,GLNT_ = 6.83 × 10^−5^ K_21,ATP_ = 6.59 × 10^−6^ K_21,EGLN_ = 1.14 × 10^−8^
22	${{\rm{V}}}_{\mathrm{GluT}}={{\rm{V}}}_{\max ,\mathrm{GLUT}}\frac{\mathrm{GLU}}{\mathrm{GLU}+{{\rm{K}}}_{22,\mathrm{GLU}}}\frac{\mathrm{ADP}}{\mathrm{ADP}+{{\rm{K}}}_{22,\mathrm{ADP}}}$	V_max,GluT_ = 2.90 × 10^−2^ K_22,ADP_ = 3.05 × 10^−6^ K_22,GLU_ = 4.43 × 10^−1^
23	${{\rm{V}}}_{\mathrm{GLNase}}={{\rm{V}}}_{\max ,\mathrm{GLNase}}\frac{\mathrm{GLN}}{\mathrm{GLN}+{{\rm{K}}}_{23,\mathrm{GLN}}}$	V_max,GLNase_ = 1.27 × 10^−3^ K_23,GLN_ = 7.16 × 10^−1^
24	${{\rm{V}}}_{\mathrm{GLDH}}={{\rm{V}}}_{\max ,\mathrm{GLDH}}\frac{\mathrm{GLU}}{\mathrm{GLU}+{{\rm{K}}}_{24,\mathrm{Glu}}}\frac{\mathrm{NAD}}{\mathrm{NAD}+{{\rm{K}}}_{24,\mathrm{NAD}}}$	V_max,GLDH_ = 1.21 × 10^−6^ K_24,GLU_ = 3.51 × 10^−3^ K_24,NAD_ = 10^−8^
25	${{\rm{V}}}_{\mathrm{ASTA}}={{\rm{V}}}_{\max ,\mathrm{ASTA}}\frac{\mathrm{OAA}}{\mathrm{OAA}+{{\rm{K}}}_{25,\mathrm{OAA}}}\frac{\mathrm{GLU}}{\mathrm{GLU}+{{\rm{K}}}_{25,\mathrm{GLU}}}$	V_max,ASTA_ = 10^−6^ K_25,GLU_ = 10^−5^ K_25,OAA_ = 10^−12^
26	${{\rm{V}}}_{\mathrm{AspT}}={{\rm{V}}}_{\max ,\mathrm{ASPT}}\frac{\mathrm{EASP}}{\mathrm{EASP}+{{\rm{K}}}_{26,\mathrm{EASP}}}\frac{\mathrm{ATP}}{\mathrm{ATP}+{{\rm{K}}}_{26,\mathrm{ATP}}}$	V_max,ASPT_ = 7.01 × 10^−5^ K_26,ATP_ = 10^−12^ K_26,EASP_ = 6.85
27	${{\rm{V}}}_{\mathrm{AsnT}}={{\rm{V}}}_{\max ,\mathrm{ASNT}}\frac{\mathrm{EASN}}{\mathrm{EASN}+{{\rm{K}}}_{27,\mathrm{EASN}}}\frac{\mathrm{ATP}}{\mathrm{ATP}+{{\rm{K}}}_{27,\mathrm{ATP}}}$	V_max,ASNT_=3.23 × 10^−6^ K_27,ATP_ = 10^−12^ K_27,EASN_ = 4.97 × 10^−8^
28	${{\rm{V}}}_{\mathrm{AsnS}}={{\rm{V}}}_{\max ,\mathrm{ASNS}}\frac{\mathrm{ASP}}{\mathrm{ASP}+{{\rm{K}}}_{28,\mathrm{ASP}}}\frac{\mathrm{ATP}}{\mathrm{ATP}+{{\rm{K}}}_{28,\mathrm{ATP}}}$	V_max,ASNS_ = 1.10 × 10^−5^ K_28,ATP_ = 10^−12^ K_28,ASP_ = 1.46 × 10^−5^
29	${{\rm{V}}}_{\mathrm{Asnase}}={{\rm{V}}}_{\max ,\mathrm{ASNase}}\frac{\mathrm{ASN}}{\mathrm{ASN}+{{\rm{K}}}_{29,\mathrm{ASN}}}$	V_max,ASNase_ = 2.32 × 10^−2^ K_29,ASN_ = 7.36 × 10^−1^
30	${{\rm{V}}}_{\mathrm{ArgT}}={{\rm{V}}}_{\max ,\mathrm{ARGT}}\frac{\mathrm{EARG}}{\mathrm{EARG}+{{\rm{K}}}_{30,\mathrm{EARG}}}\frac{\mathrm{ATP}}{\mathrm{ATP}+{{\rm{K}}}_{30,\mathrm{ATP}}}$	V_max,ARGT_ = 3.84 × 10^−3^ K_30,ATP_ = 10^−12^ K_30,EARG_ = 2.42 × 10^2^
31	${{\rm{V}}}_{\mathrm{ASL}}={{\rm{V}}}_{\max ,\mathrm{ASL}}\frac{\mathrm{AS}}{\mathrm{AS}+{{\rm{K}}}_{31,\mathrm{AS}}}$	V_max,ASL_ = 2.00 × 10^−6^ K_31,AS_ = 10^−5^
32	${{\rm{V}}}_{\mathrm{Arg}1}={{\rm{V}}}_{\max ,\mathrm{ARG}1}\frac{\mathrm{ARG}}{\mathrm{ARG}+{{\rm{K}}}_{32,\mathrm{ARG}}}$	V_max,ARG1_=4.00 × 10^−6^ K_32,ARG_ = 10^−4^
33	${{\rm{V}}}_{\mathrm{OTC}}={{\rm{V}}}_{\max ,\mathrm{OTC}}\frac{\mathrm{CP}}{\mathrm{CP}+{{\rm{K}}}_{33,\mathrm{CP}}}\frac{\mathrm{ORN}}{\mathrm{ORN}+{{\rm{K}}}_{33,\mathrm{ORN}}}$	V_max,OTC_ = 1.10 × 10^−6^ K_33,CP_ = 10^−12^ K_33,ORN_ = 10^−5^
34	${{\rm{V}}}_{\mathrm{iNOS}}={{\rm{V}}}_{\max ,\mathrm{iNOS}}\frac{\mathrm{ARG}}{\mathrm{ARG}+{{\rm{K}}}_{34,\mathrm{ARG}}}\frac{\mathrm{NADP}}{\mathrm{NADP}+{{\rm{K}}}_{34,\mathrm{NADP}}}$	V_max,iNOS_ = 10^−6^ K_34,ARG_ = 10^−5^ K_34,NADP_ = 10^−12^
35	${{\rm{V}}}_{\mathrm{ASS}}={{\rm{V}}}_{\max ,\mathrm{ASS}}\frac{\mathrm{CTR}}{\mathrm{CTR}+{{\rm{K}}}_{35,\mathrm{CTR}}}\frac{\mathrm{ASP}}{\mathrm{ASP}+{{\rm{K}}}_{35,\mathrm{ASP}}}\frac{\mathrm{ATP}}{\mathrm{ATP}+{{\rm{K}}}_{35,\mathrm{ATP}}}$	V_max,ASS_ = 3.00 × 10^−6^ K_35,ATP_ = 10^−12^ K_35,ASP_ = 10^−5^ K_35,CTR_ = 10^−5^
36	${{\rm{V}}}_{\mathrm{CPS}}={{\rm{V}}}_{\max ,\mathrm{CPS}}\frac{{\mathrm{NH}}_{4}}{{\mathrm{NH}}_{4}+{{\rm{K}}}_{36,{\mathrm{NH}}_{4}}}\frac{\mathrm{ATP}}{\mathrm{ATP}+{{\rm{K}}}_{36,\mathrm{ATP}}}$	V_max,CPS_ = 2.43 × 10^−6^ K_36,ATP_ = 10^−12^ K_36,NH4_ = 4.13 × 10^−8^
37	${{\rm{V}}}_{\mathrm{PrAMP}}={{\rm{V}}}_{\max ,\mathrm{PrAMP}}\frac{{\rm{R}}5{\rm{P}}}{{\rm{R}}5{\rm{P}}+{{\rm{K}}}_{37,{\rm{R}}5{\rm{P}}}}$	V_max,PrAMP_ = 6.11 × 10^−7^ K_37,R5P_ = 8.90 × 10^−^10^−7^
38	${{\rm{V}}}_{\mathrm{CK}}={{\rm{V}}}_{\max ,\mathrm{CK}}^{{\rm{f}}}\frac{\mathrm{PCr}}{\mathrm{PCr}+{{\rm{K}}}_{38,\mathrm{PCr}}}\frac{\mathrm{ADP}}{\mathrm{ADP}+{{\rm{K}}}_{38,\mathrm{ADP}}}-{{\rm{V}}}_{\max ,\mathrm{CK}}^{{\rm{r}}}\frac{\mathrm{Cr}}{\mathrm{Cr}+{{\rm{K}}}_{38,\mathrm{Cr}}}\frac{\mathrm{ATP}}{\mathrm{ATP}+{{\rm{K}}}_{38,\mathrm{ATP}}}$	V^f^_max,CK_ = 10^−6^ V^r^_max,CK_ = 10^−7^ K_38,ADP_ = 10^−12^ K_38,ATP_ = 10^−12^ K_38,PCr_ = 10^−5^ K_38,Cr_ = 10^−7^
39	${{\rm{V}}}_{\mathrm{NADPHox}}={{\rm{V}}}_{\max ,\mathrm{NADPHox}}\frac{\mathrm{NADPH}}{\mathrm{NADPH}+{{\rm{K}}}_{39,\mathrm{NADPH}}}$	V_max,NADPHox_ = 1.39 × 10^−4^ K_39,NADPH_ = 6.49 × 10^−7^
40	${{\rm{V}}}_{\mathrm{leak}}={{\rm{V}}}_{\max ,\mathrm{leak}}\frac{\mathrm{NADH}}{\mathrm{NADH}+{{\rm{K}}}_{40,\mathrm{NADH}}}$	V_max,leak_ = 2.95 × 10^−3^ K_40,NADH_ = 10^−5^
41	${{\rm{V}}}_{\mathrm{Resp}}={{\rm{V}}}_{\max ,\mathrm{Resp}}\frac{\mathrm{NADH}}{\mathrm{NADH}+{{\rm{K}}}_{41,\mathrm{NADH}}}\frac{\mathrm{ADP}}{\mathrm{ADP}+{{\rm{K}}}_{41,\mathrm{ADP}}}$	V_max,Resp_ = 5.00 × 10^−5^ K_41,ADP_ = 10^−12^ K_41,NADH_ = 10^−5^
42	${{\rm{V}}}_{\mathrm{ATPase}}={{\rm{V}}}_{\max ,\mathrm{ATPase}}^{{\rm{f}}}\frac{\mathrm{ATP}}{\mathrm{ATP}+{{\rm{K}}}_{42,\mathrm{ATP}}}-{{\rm{V}}}_{\max ,\mathrm{ATPase}}^{{\rm{r}}}\frac{\mathrm{ADP}}{\mathrm{ADP}+{{\rm{K}}}_{42,\mathrm{ADP}}}$	V^f^_max,ATPase_ = 1.29 × 10^−3^ V^r^_max,ATPase_ = 8.51 × 10^−1^ K_42,ADP_ = 5.11 × 10^−2^ K_42,ATP_ = 2.04 × 10^−5^
43	${{\rm{V}}}_{\mathrm{AK}}={{\rm{V}}}_{\max ,\mathrm{AK}}^{{\rm{f}}}\frac{\mathrm{AMP}}{\mathrm{AMP}+{{\rm{K}}}_{43,\mathrm{AMP}}}\frac{\mathrm{ATP}}{\mathrm{ATP}+{{\rm{K}}}_{43,\mathrm{ATP}}}-{{\rm{V}}}_{\max ,\mathrm{AK}}^{{\rm{r}}}\frac{\mathrm{ADP}}{\mathrm{ADP}+{{\rm{K}}}_{43,\mathrm{ADP}}}$	V^f^_max,AK_ = 1.55 × 10^−5^ V^r^_max,AK_ = 2.31 × 10^−6^ K_43,ADP_ = 2.68 × 10^−8^ K_43,AMP_ = 8.31 × 10^−10^ K_43,ATP_ = 6.18 × 10^−8^
44	${{\rm{V}}}_{\mathrm{NAT}}={{\rm{V}}}_{\max ,\mathrm{NAT}}\frac{{\rm{R}}5{\rm{P}}}{{\rm{R}}5{\rm{P}}+{{\rm{K}}}_{44,{\rm{R}}5{\rm{P}}}}\frac{{\rm{X}}5{\rm{P}}}{{\rm{X}}5{\rm{P}}+{{\rm{K}}}_{44,{\rm{X}}5{\rm{P}}}}\frac{\mathrm{EG}\mathrm{ln}}{\mathrm{Cr}+{{\rm{K}}}_{44,\mathrm{EG}\mathrm{ln}}}\frac{\mathrm{ATP}}{\mathrm{ATP}+{{\rm{K}}}_{44,\mathrm{ATP}}}$	V_max,NAT_ = 1.81 × 10^−7^ K_44,R5P_ = 10^−12^ K_44,X5P_ = 1.12 × 10^−5^ K_44,EGln_ = 1.02 × 10^−2^ K_44,ATP_ = 5.12 × 10^−6^
45	${{\rm{V}}}_{\mathrm{NHG}}={{\rm{V}}}_{\max ,\mathrm{NHG}}^{{\rm{f}}}\frac{\mathrm{NAD}}{\mathrm{NAD}+{{\rm{K}}}_{45,\mathrm{NAD}}}\frac{\mathrm{ATP}}{\mathrm{ADP}+{{\rm{K}}}_{45,\mathrm{ATP}}}-{{\rm{V}}}_{\max ,\mathrm{NHG}}^{{\rm{r}}}\frac{\mathrm{NADP}}{\mathrm{Cr}+{{\rm{K}}}_{45,\mathrm{NADP}}}\frac{\mathrm{ADP}}{\mathrm{ADP}+{{\rm{K}}}_{45,\mathrm{ADP}}}$	V^f^_max,NHG_ = 4.19 × 10^−7^ V^r^_max,NHG_ = 1.84 × 10^−8^ K_45,ADP_ = 10^−6^ K_45,ATP_ = 1.05 × 10^−5^ K_45,NAD_ = 10^−6^ K_45,NADP_ = 10^−6^
46	${{\rm{V}}}_{\mathrm{growth}}={{\rm{V}}}_{\max ,\mathrm{growth}}\frac{{\rm{G}}6{\rm{P}}}{{\rm{G}}6{\rm{P}}+{{\rm{K}}}_{46,{\rm{G}}6{\rm{P}}}}\frac{{\rm{R}}5{\rm{P}}}{{\rm{R}}5{\rm{P}}+{{\rm{K}}}_{46,{\rm{R}}5{\rm{P}}}}\frac{\mathrm{EGLN}}{\mathrm{EGLN}+{{\rm{K}}}_{46,\mathrm{EGLN}}}\frac{\mathrm{ATP}}{\mathrm{ATP}+{{\rm{K}}}_{46,\mathrm{ATP}}}$	V_max,growth_ = 4.23 × 10^−3^ K_46,EGLN_ = 3.20 × 10^−1^ K_46,G6P_ = 10^−12^ K_46,R5P_ = 10^−12^ K_46,ATP_ = 10^−12^

V_max_ in mmol.10^−6^ cells.h^−1^ and and K_m_ in mmol.10^−6^ cells and in mM for intracellular and extracellular concentrations respectively.

**Table 3:** Metabolites initial concentration.

Metabolite	Initial concentration		Metabolite	Initial concentration	
ACCoA	1.54 × 10^−8^	mmol 10^−6^ cells	F6P	10^−7^	mmol 10^−6^ cells
ADP	3.74 × 10^−7^	mmol 10^−6^ cells	FUM	10^−6^	mmol 10^−6^ cells
AKG	6.85 × 10^−7^	mmol 10^−6^ cells	G6P	10^−8^	mmol 10^−6^ cells
ALA	10^−6^	mmol 10^−6^ cells	GAP	4 × 10^−8^	mmol 10^−6^ cells
AMP	8.25 × 10^−8^	mmol 10^−6^ cells	Gln	4 × 10^−3^	mmol 10^−6^ cells
ARG	1.4 × 10^−3^	mmol 10^−6^ cells	Glu	5.625 × 10^−4^	mmol 10^−6^ cells
AS	10^−8^	mmol 10^−6^ cells	MAL	4.1 × 10^−7^	mmol 10^−6^ cells
ASN	6.45 × 10^−4^	mmol 10^−6^ cells	NAD+	10^−6^	mmol 10^−6^ cells
ASP	3.83 × 10^−4^	mmol 10^−6^ cells	NADH	10^−6^	mmol 10^−6^ cells
ATP	2.57 × 10^−6^	mmol 10^−6^ cells	NADP+	2 × 10^−6^	mmol 10^−6^ cells
CIT	2 × 10^−7^	mmol 10^−6^ cells	NADPH	1.26 × 10^−7^	mmol 10^−6^ cells
CO_2_	2 × 10^−5^	mmol 10^−6^ cells	NH4	0.1287	mmol 10^−6^ cells
CP	10^−8^	mmol 10^−6^ cells	OAA	1.5 × 10^−8^	mmol 10^−6^ cells
Cr	3.9 × 10^−5^	mmol 10^−6^ cells	ORN	10^−8^	mmol 10^−6^ cells
CTR	10^−8^	mmol 10^−6^ cells	PCr	2 × 10^−5^	mmol 10^−6^ cells
EARG	0.669	mmol L^−1^	PEP	1.25 × 10^−7^	mmol 10^−6^ cells
EASN	0.313	mmol L^−1^	PYR	3.97 × 10^−5^	mmol 10^−6^ cells
EASP	0.184	mmol L^−1^	R5P	10^−7^	mmol 10^−6^ cells
EGLC	9.843	mmol L^−1^	SCoA	4 × 10^−7^	mmol 10^−6^ cells
EGLN	2.07	mmol L^−1^	SUC	3.3345 × 10^−6^	mmol 10^−6^ cells
EGLU	0.27	mmol L^−1^	X5 P	9.25 × 10^−8^	mmol 10^−6^ cells
ELAC	0.5033	mmol L^−1^	X	0.2	10^6^ cells mL^−1^
ENH4	0.12	mmol L^−1^			

Using the parameter values determined in a previous work on CHO cells^11^ (and from references therein), a first sensitivity analysis was performed on the resulting model, aiming to identify the most critical (i.e. sensitive) parameters. In brief (see the Supplementary material for details), because of the large number of parameter values to be determined, an iterative procedure including manual trials on sub-groups of parameters and a model simulation global error minimization step, allowed identifying optimal model parameter values. Sensitivity analysis of model parameters on the model acuity to simulate experimental reality was performed as follow. Values of model parameters were changed by ±5 or 10%, once at a time, from their optimal value, and the normalized sum-squared differences were calculated as described below. A model sensitivity analysis on initial conditions has also been performed to evaluate the effect of measurement errors. ${X}^{{mea}}$ and ${X}^{{sim}}$ are respectively the experimental data and simulated values for each state variable $m$ and time $k$, and the weight is the inverse of the variance of the experimental data for each state variable, ${{var}}_{m}^{-1}$.

1 $$\min \,WSSRES=\,[\sum _{t=1}^{k}\sum _{m=1}^{n}{({X}_{t,m}^{sim}-{X}_{t,m}^{mea})}^{2}va{r}_{m}^{-1}]$$

Finally, the 95% confidence intervals on the estimated model parameters were computed, using the Matlab functions “nlinfit.m”, “nlparci.m” and “nlpredci.m”. Following the parameter estimation step (“nlinfit.m”), the confidence intervals were computed for the estimated model parameters (“nlparci.m”). The 30 most sensitive parameters (Table 4) have been used in the computation of the confidence intervals.

**Table 4:** Model sensitive parameter optimal values and confidence intervals.

Model parameter	Optimal value minimizing simulation error	95% Confidence intervals	
		Lower bound	Upper bound
V_max,HK_	5.08 × 10^−4^	4.92E-04	5.79E-4
V^f^_max,PGI_	3.53 × 10^−4^	1.62E-04	4.43E-04
V_max,G6PDH_	7.62 × 10^−5^	6.12E-05	7.38E-05
V_max,EP_	6.74 × 10^−5^	3.81E-05	1.09E-04
V_max,TK_	3.37 × 10^−5^	1.39E-05	4.28E-05
V_max,PFK_	3.42 × 10^−4^	3.12E-04	3.96E-04
V_max,PK_	9.79 × 10^−4^	9.70E-04	1.00E-03
V_max,CITS_	9.33 × 10^−6^	9.06E-06	1.14E-05
V_AKGDH_	1.14 × 10^−5^	1.14E-05	1.30E-05
V_max,FUM_	5.03 × 10^−6^	4.61E-06	5.10E-06
V_max,PC_	1.23 × 10^−5^	1.06E-05	2.15E-05
V_max,ASNS_	1.10 × 10^−5^	1.10E-05	1.10E-05
V_max,ASNase_	2.32 × 10^−2^	9.97E-03	2.79E-02
V_max,OTC_	1.10 × 10^−6^	9.77E-07	1.28E-06
V_max,iNOS_	10^−6^	8.90E-07	1.16E-06
V_max,ASS_	3.00 × 10^−6^	2.94E-06	3.07E-06
V_max,PrAMP_	6.11 × 10^−7^	6.02E-07	6.19E-07
V^f^_max,AK_	1.55 × 10^−5^	1.53E-05	1.56E-05
V^r^_max,AK_	2.31 × 10^−6^	2.27E-06	2.49E-06
V^f^_max,ATPase_	1.29 × 10^−3^	8.38E-04	1.53E-03
V_max,NADPHox_	1.39 × 10^−4^	1.29E-04	1.35E-04
V_max,growth_	4.23 × 10^−3^	4.23E-03	4.42E-03
K_1,ATP_	1.08 × 10^−5^	1.05E-05	1.38E-05
K_4,R5P_	10^−6^	3.19E-07	2.00E-06
K_5,R5P_	10^−6^	4.95E-08	1.42E-06
K_28,ASP_	1.46 × 10^−5^	1.41E-05	1.51E-05
K_29,ASN_	7.36 × 10^−1^	6.91E-01	7.82E-01
K_35,CTR_	10^−5^	9.21E-06	1.07E-05
K_37,R5P_	8.90 × 10^−7^	8.66E-07	9.15E-07
K_42,ATP_	2.04 × 10^−5^	1.03E-05	2.55E-05

**(**V_max_ in mmol.L^−1^.h^−1^ and and K_m_ in mM).

## Results and Discussion

### The Kinetic-Metabolic Model Simulates MDSCs Metabolic-Time Profile

**Figure 2:**
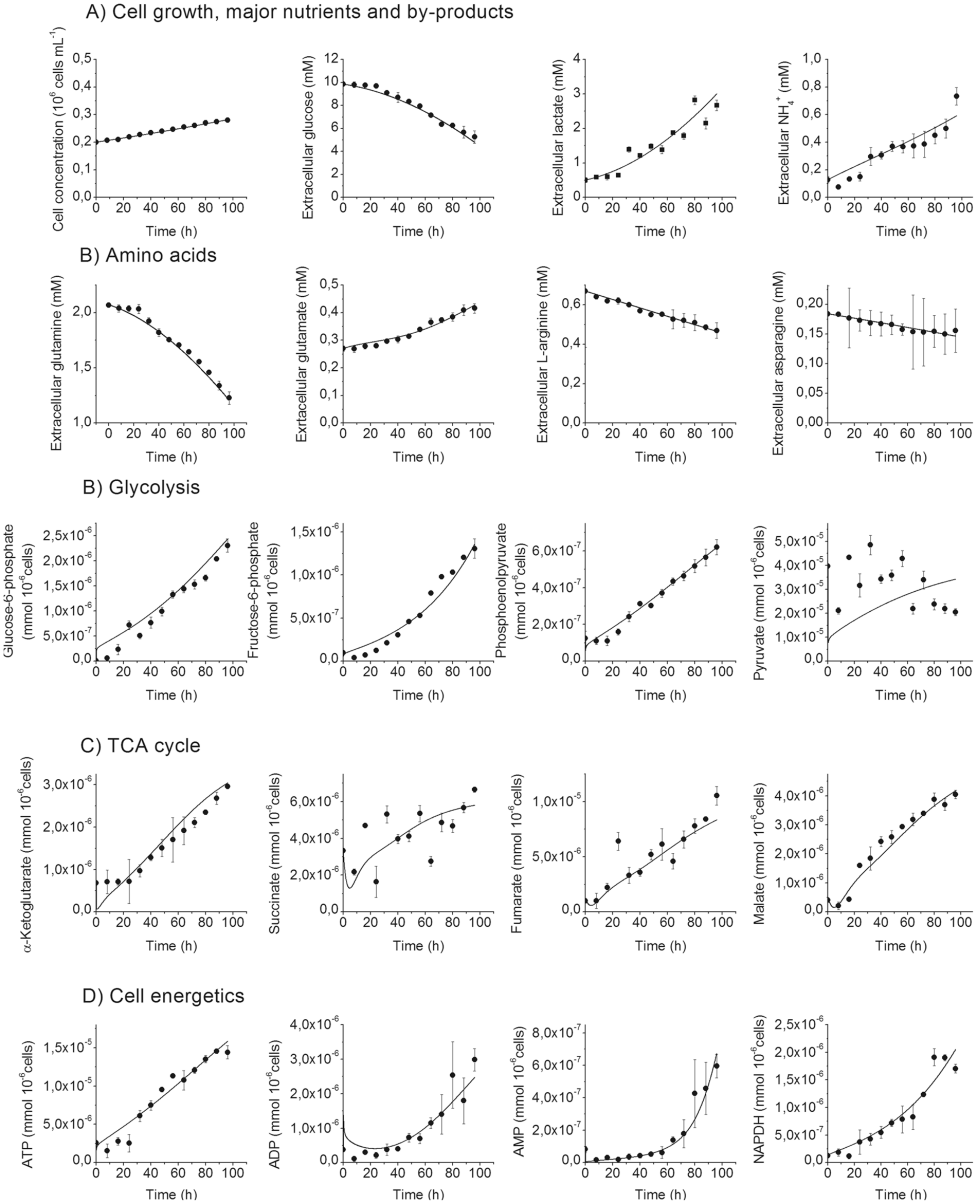
Simulated (line) and experimental data (dots) for BM-derived MDSCs. (**A**) Cell growth and nutritional profile. (**B**) Glycolysis. (**C**) TCA cycle. (**D**) Cell energetics. Error bars are calculated based on independent triplicates. Cell density is in 10^6^ cells.mL^−1^ and intracellular and extracellular concentrations are in mmol.10^−6^ cells and mM, respectively.

The resulting metabolic model has then been used to simulate metabolites concentration with time, supported (Fig. 2) or not (Fig. S2) with experimental data. The time-evolution of metabolites concentration were thus either compared to experimental data (when available) and to range of values found in literature, and thus confirmed the biologic relevance of the set of parameters as well as of the metabolic model structure.

**Figure 3:**
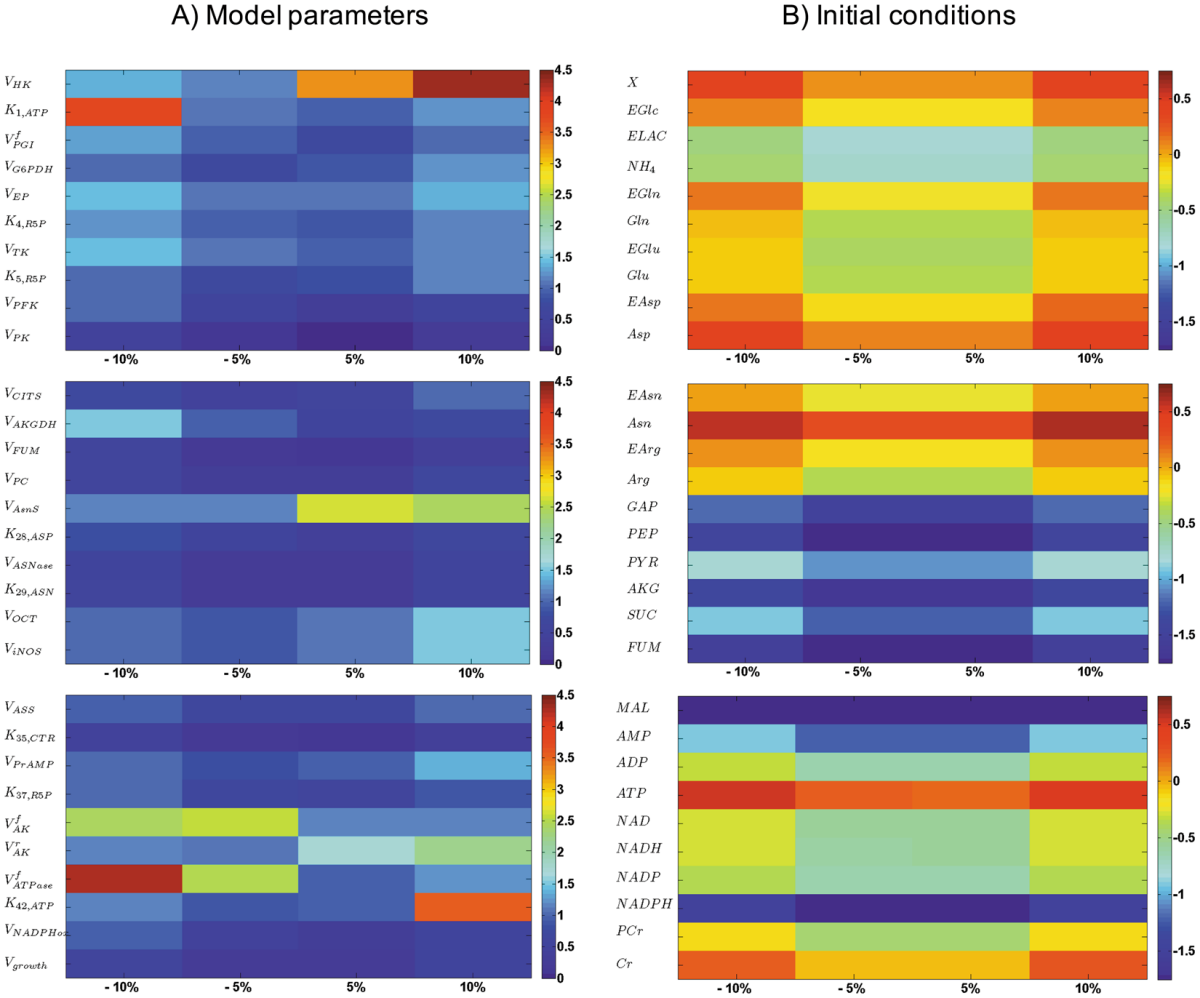
Sensitivity analysis of the model. Analysing most sensitive (**A**) parameters and (**B**) initial conditions. The computed value is log WSSRES and the distance is among −10%, −5%, + 5% and + 10%.

The sensitivity analysis (Fig. 3A) procedure allowed to rank the parameters from their decreasing influence, and to remove parameters that were not contributing to model sensitivity from further optimization cycles, keeping them at their optimal values. Only the sensitivity analysis results for the final model (i.e. no more improvement of the simulation error) are shown here. Interestingly, most of the parameters (102 on 131) reveal to be non-sensitive with a WSSRES value lower than 0.1. This lack of sensitivity may partially come from the large number of parameters to be optimized, the low number of experimental data, and the important error bars. These non-sensitive parameters are within biologically relevant values and describe existing active pathways and enzymatic reactions, but they may require an expanded experimental space out of the current one to be solicited. Moreover and of interest, we have previously showed that the sensitive parameters are the ones needing to be changed while describing a modified genetic expression^10, 11^. Taking all of the above, the model structure was not reduced.

Specifically, the model reveals to be primarily sensitive to parameters of glycolysis and energetic reactions, partially to TCA cycle and pentose phosphate pathway, and to a lesser extent to urea cycle and amino acids catabolism. The maximum specific glucose uptake rate (${\nu }_{{\max }{HK}}$) and the maximal ATPase (${\nu }_{{\max }{ATPase}}$) reaction rate as well as its affinity constant (${K}_{42,{ATP}}$) showed to strongly affect simulation error. This result is particularly interesting since cell energetics plays a major role on the regulation of central carbon metabolism fluxes^18^. In complement, we also observed that the model is less sensitive to errors on initial conditions (Table 3) (Fig. 3B), only showing here the ones with the highest effect on the global error. This result is of interest since this type of dynamic model only requires initial conditions, together with kinetic parameters, to simulate a cell population behavior with time. Indeed, it is interesting to note that the high simplification level of the proposed model is not resulting in high level of simultation errors when challenged from either parameters value or initial concentration conditions. Expanding the amount of biochemical reactions would require, in a further work, expanding the amount of quantified metabolites to support the according increase of model parameters. The model has thus been used to perform a dynamic metabolic flux analysis (dMFA), analysing the results as new data per se as well as to further validate model simulations validity.

### MDSCs Exhibit Warburg Effect During their Maturation Process

**Figure 4:**
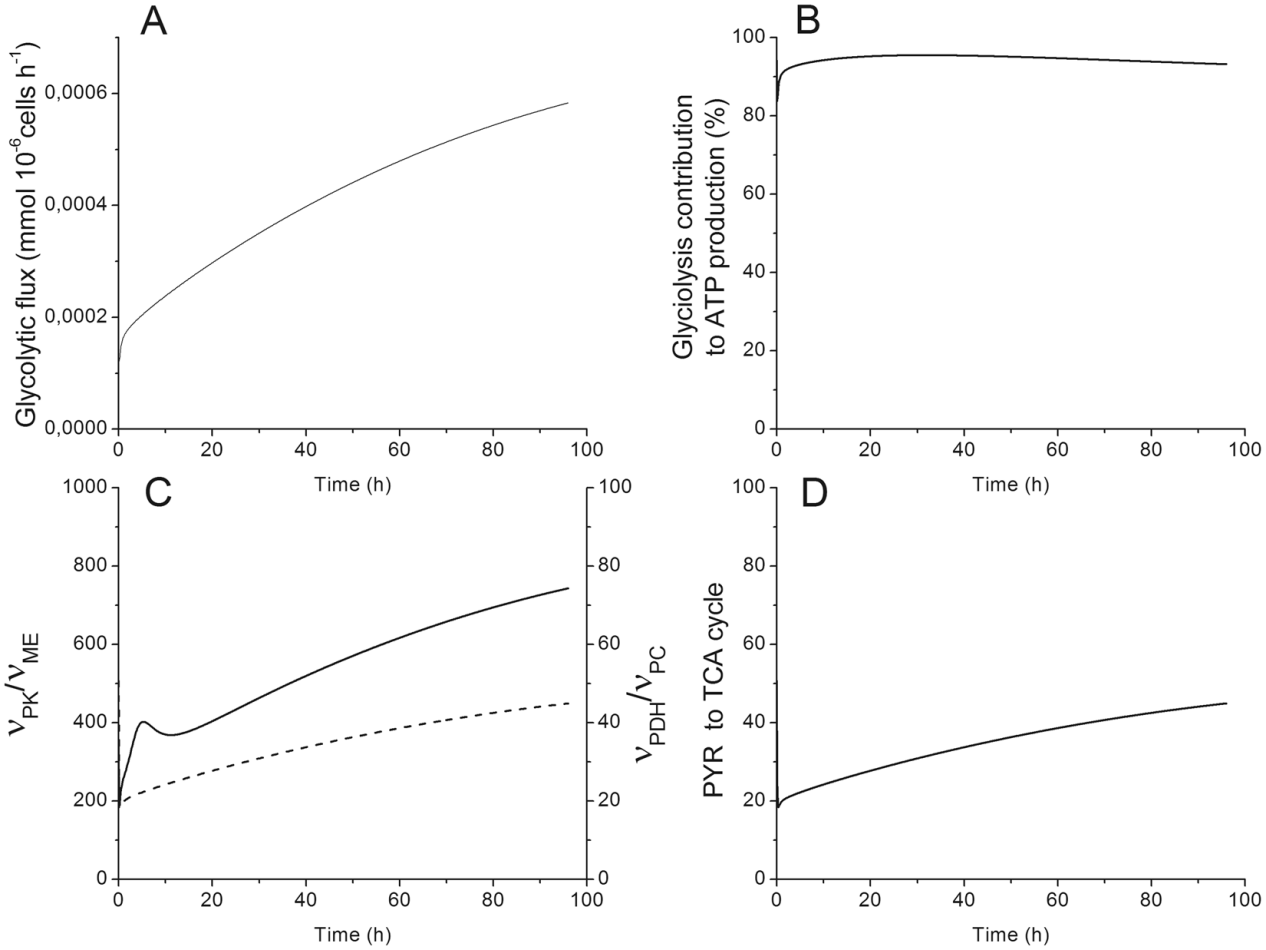
Time-evolution of glycolysis. (**A**) Glycolytic flux (${\nu }_{{PK}}$) in ${mmol}.{10}^{-6}{cells}.{h}^{-1}$. (**B**) Glycolysis contribution to ATP production $({v}_{{PGK}}+{v}_{{PK}})/({v}_{{PGK}}+{v}_{{PK}}+{v}_{{SCOAS}}+4{v}_{{resp}}+{v}_{{GluT}}+{v}_{{CK}}^{f}+{v}_{{AK}}^{r}+{v}_{{ATPase}}^{r})$. (**C**) Comparison of pyruvate producing (${v}_{{PK}}/{v}_{{ME}}$) and consuming (${v}_{{PDH}}/{v}_{{PC}}$) fluxes. (**D**) Ratio of pyruvate entering the TCA cycle (${v}_{{PDH}}/({v}_{{PK}}+{v}_{{ME}})$). All results obtained from the same model simulation than that of Fig. 2.

Dynamic metabolic flux analysis confirms MDSCs mimick tumour cells metabolic profile. Indeed, model simulation of experimental data confirm that BM-derived MDSCs exhibit a high glycolytic activity level as revealed by the continuous increase of the flux through pyruvate kinase (Fig. 4A). Moreover, up to 95% of the ATP generated in MDSCs are glycolysis-dependent (Fig. 4B), with thus an expected reduced oxygen consumption rate. This result correlates with previous findings where BM-derived MDSCs were shown to decrease their oxygen consumption, and consequently the oxidative phosphorylation -dependent ATP production, by 60% during their maturation process^8^. This behavior is typical to tumor cells. In fact, tumour cells are known to be metabolically heterogeneous^12, 13^ with a high glycolytic metabolism and a reduced, but not completely suppressed, oxidative phosphorylation^21^. A high aerobic metabolism was also shown to support highly proliferative tumors cells whereas a less efficient electron transfer chain in mitochondria is associated to ROS production^22^. This result is of utmost interest since despite the abundance of oxygen in *in vitro* culture condition, a glycolytic metabolism is favored in BM-derived MDSCs, a behaviour reported in some tumour cells. This metabolic profile may represent another mechanism of immunosuppression. In fact, in the tumour microenvironment, anti-tumour immune effector cells will compete with tumour cells and MDSCs that both exhibit high glucose uptake rates. Whereas, immune cells do not have any metabolic elasticity to acclimatize to low oxygen tension and limited glucose availability, and these conditions may trigger immune cell dysfunction and death, which can indirectly lead to tumour escape and progression. In a previous work on BM-derived MDSCs and also on a MSC-1 immortalized cell line^8^, we observed several similarities between MDSCs and tumor cells metabolic behavior, such as a glycolytic metabolism, high glucose and glutamine uptake rates, reduced oxygen consumption rate but a high TCA cycle activity. It is known that MDSCs are getting fully activated and matured in the tumor microenvironment by means of tumor-derived factors (TDFs such as GM-CSF, TGF, IL-6, etc). These TDFs indirectly induce a similar metabolic behavior to tumor cells and this is further confirmed in this work from the dynamic metabolic flux analysis (dMFA) performed using our model.

### Most of the Glucose-Derived Carbon Enters the TCA Cycle

**Figure 5:**
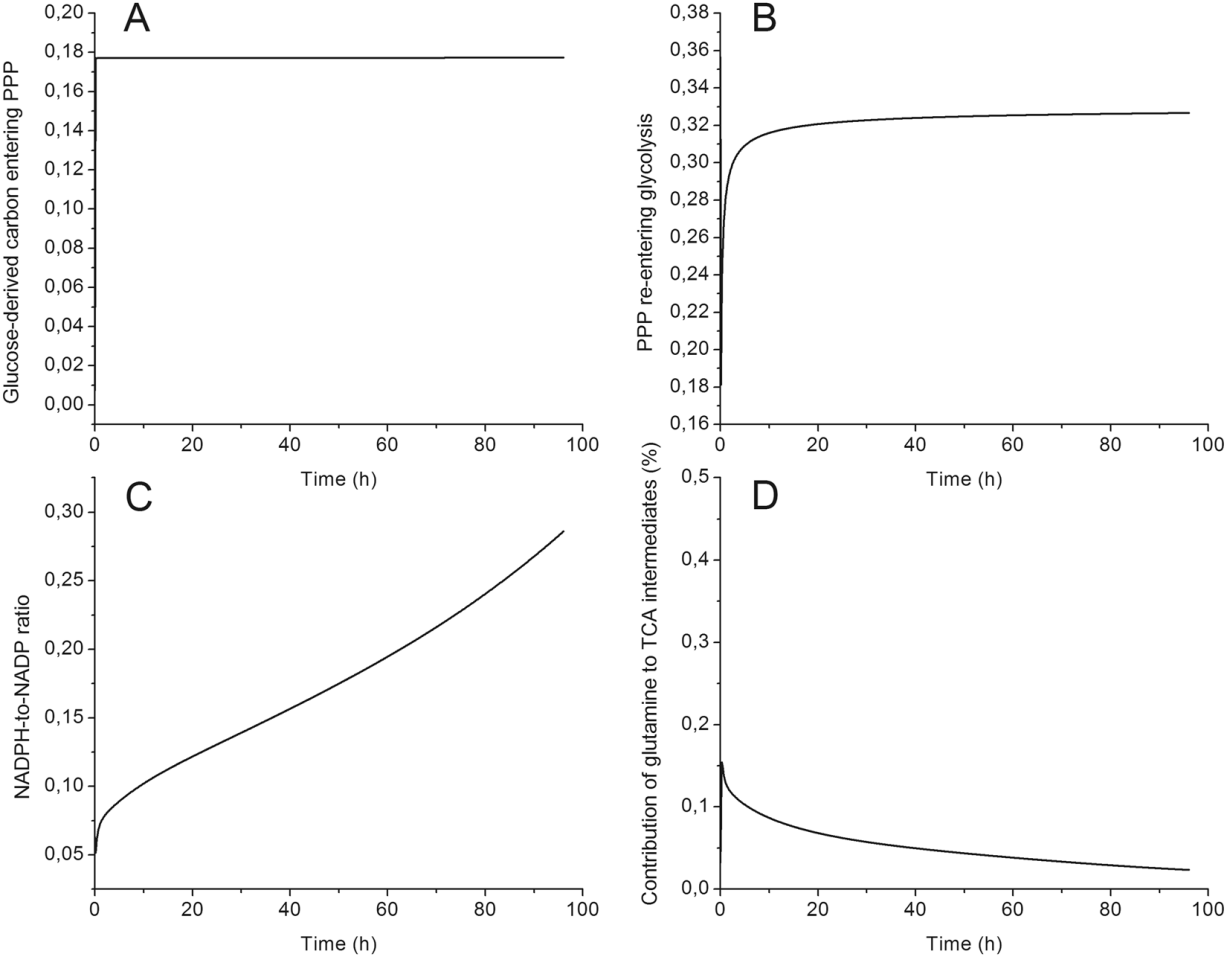
Pentose phosphate pathway and glutaminolysis modulation in BM-derived MDSCs. (**A**) G-6-P branch point (${v}_{G6{PDH}}/{v}_{{HK}}$). (**B**) Recirculation of glucose-derived carbon from pentose phosphate pathway into glycolysis (${v}_{{TK}}/{v}_{G6{PDH}}$). (**C**) NADPH-to-NADP ratio. (**D**) Contribution of L-glutamine to TCA cycle intermediates replenishment (${v}_{{GLDH}}/({v}_{{GLDH}}+{v}_{{PC}}+{v}_{{PDH}}+{v}_{{AlaTA}}^{f}+{v}_{{AspAT}}^{r})$). All results obtained from the same model simulation than that of Fig. 2.

A dMFA study at the pyruvate branch point revealed that pyruvate is mainly produced through the pyruvate kinase enzyme, with a flux of ~700-fold than that through the malic enzyme. This result suggests that pyruvate is principally derived from glucose than recirculated from the TCA cycle (Fig. 4C). Likewise, the comparison of the fluxes allowing the entry of glucose-derived carbon into the TCA cycle showed that the flux through pyruvate dehydrogenase was higher (up to 50-fold), and kept increasing with time, than the flux through pyruvate carboxylase (Fig. 4C). Moreover, the percentage of glucose-derived carbon entering the TCA cycle was increasing (from 50 to 80%) during the BM-derived MDSC maturation process (Fig. 4D). These anaplerotic/cataplerotic reactions are known to be important to the replenishment of TCA cycle intermediates^23^. The high glycolysis-TCA cycle activity is probably associated to the acquisition of the immunosuppressive phenotype, and the maintenance of inflammatory environment at the tumour edge, by supporting enzyme and surface markers expression, cytokine/chemokine synthesis, etc. In addition, some of the TCA cycle intermediates have roles other than being a carbon source. For instance, the accumulation of fumarate and succinate stabilizes hypoxia induction factor 1α^22, 24^ that was shown to be expressed in MDSCs to adapt to low oxygen tension in tumour^25^. Since BM-derived MDSCs exhibit a low growth rate during the 96 hours of the study, a longer culture period or the analysis of flux distribution in *in vivo* generated MDSCs is required to confirm if this behavior, i.e. high TCA cycle activity, is associated to proliferative purposes as previously reported in cancer cells^22^. Moreover, the analysis of the G6P branch point shows that only 20% of the glucose-derived carbon enters the pentose phosphate pathway, since the ratio of the flux through G6PDH to the one through HK was kept constant at 20% (Fig. 5A). A total of 30% of the carbon that enters the pentose phosphate pathway is recirculated to glycolysis (Fig. 5B). This metabolic behavior occurs when both NADPH and ATP are needed, but only sparsely ribose-5-phosphate, which is coherent with the low growth rate observed^8^. Glycolytic carbons are then shunted into the oxidative phase of the pentose phosphate pathway, and consequently in the non-oxidative phase leading to re-entering glycolysis^26^. This was confirmed by the NADPH-to-NADP ratio (Fig. 5C) which increased (up to 9) rapidly for the last 30 hours, where BM-derived MDSCs were fully mature and active. Furthermore, as we discussed above, glycolysis was mainly responsible for ATP production, which is thus confirming then that MDSCs have the ability to modulate their central carbon metabolism to ensure the proper bioenergetics state required for their maturation and activity.

### MDSCs Immunosuppression Machinery is not Energetically Costly

Contrarily to glucose-derived carbon, which is mainly entering into the TCA cycle to sustain its high activity, L-glutamine (GLN in the model) only contributes sparsely to the replenishment of TCA cycle intermediates (Fig. 5D). However, the fate of GLN-derived carbon in MDSCs is ambiguous and requires further investigation using ^13^C-labelled GLN, but our results clearly show it is likely used in the endogenous synthesis of L-arginine to support cells immunosuppressive potential^27^. As BM-derived MDSCs exhibit a high GLN consumption rate, immune effector cells will also compete with MDSCs in addition to tumour cells for GLN, a phenomenon that is crucial for their proliferation.

**Figure 6:**
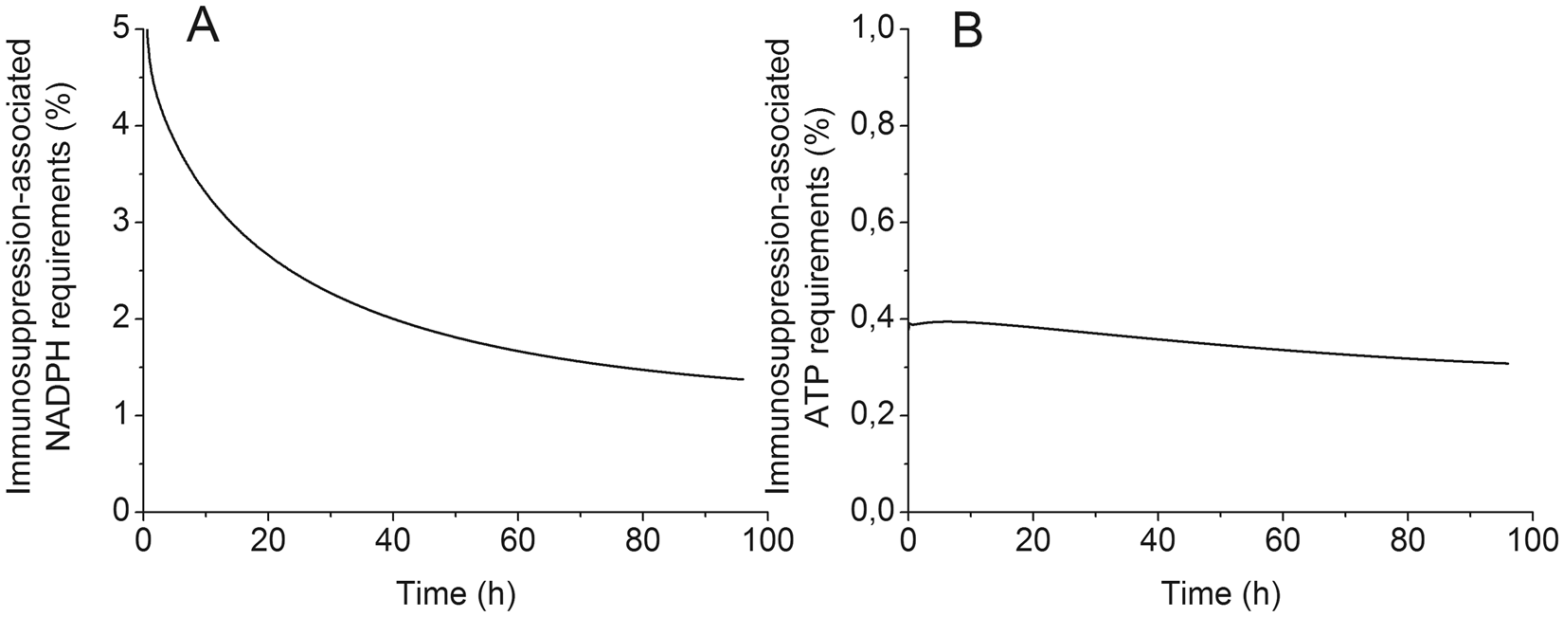
Energetics supporting MDSCs immunosuppressive activity. (**A**) Percentage of NADPH consumed by L-arginine metabolism, iNOS and ARG1 activities and L-arg uptake and endogenous synthesis. (**B**) Percentage of ATP consumed by L-arginine metabolism. All results obtained from the same model simulation than that of Fig. 2.

Interestingly, the dMFA study showed that the L-arginine metabolism (including its uptake, its metabolism by the inducible Nitric Oxide Synthase and Arginase 1 and its endogenous synthesis) do not use considerable quantities of ATP and NADPH. In fact, the percentage of NADPH used by the L-arginine metabolism is decreasing from 5 to 1% (Fig. 6A). This decrease is associated to a higher flux through NADPH oxidase during BM-derived MDSCs maturation process. However, NADPH oxidase co-produces NADP^+^ and superoxide (O_2_^−^), the latter being a precursor for the generation of immunosuppressive species ROS and RNOS^28^. A similar decreasing trend from 6 to 1% was simulated for the percentage of ATP consumed by the L-arginine metabolism (Fig. 6B). These findings support the hypothesis that L-arginine metabolism is not energetically costly but further investigation is still required to verify the energetic needs of complementary immunosuppressive mechanisms that are not modelled in this work, such as tryptophan metabolism, expression of messenger molecules, etc. Interestingly, there are only 30 sensitive parameters (Table 4) on the 136 parameters of the model, which cover all metabolic sub-networks, and especially including those that define specific functions of MDSCs.

## Conclusion

This work on model simulation of MDSCs maturation, used a wide set of experimental data for extra- and intracellular metabolites concentration to develop a descriptive and predictive dynamic metabolic model. The model then enabled performing a dMFA study, which results allowed highlighting the carbon distribution dynamics during MDSCs maturation and also in fully mature and active cells. Mainly, MDSCs revealed to be dependent on the glycolysis and the glucose-derived carbon to ensure high central carbon metabolism activity and sustained production of ATP. Pentose phosphate pathway and oxidative phosphorylation showed being active at the minimal level allowing to replenish the NADPH pool and anabolic precursors. This metabolic behaviour was not justified by specific requirements of MDSCs immunosuppression machinery neither at the carbon intermediate or energy level. However, this metabolic profile indirectly favors tumour invasiveness and shall be considered as a target for anti-cancer immunotherapy. In this work, the interpretation of carbon flux distribution simulated by the model leads to the identification of the specific metabolic signatures of mature MDSCs that are directly associated to the immunosuppression phenotype. This *in silico* platform thus allows prospecting MDSCs dynamic behaviour under stimulation or inhibition conditions, as well as it represents a complementary tool to existing tools for studying the immunosuppression phenomenon^29^. Cell metabolomic studies provide new and complementary insights enabling to better understand the immunosuppression phenomenon, and such dynamic model may be useful to the identification of novel biomarkers as well as to define therapeutic strategies to modulate this phenomenon. The validation of the predictive capacity of the model will, however, be possible with the accumulation of large experimental data sets, as well as further implementing the metabolic network. This first modelling work on MDSCs thus brings a basic framework for the study of the immunosuppression phenomenon, with the most sensitive parameters as keys of further model development.

## Electronic supplementary material

Supplementary information

## References

[1] Mahoney KM, Rennert PD, Freeman GJ (2015). Combination cancer immunotherapy and new immunomodulatory targets. Nature reviews. Drug discovery.

[2] Gabrilovich, D. I. *et al*. The terminology issue for myeloid-derived suppressor cells. *Cancer research* **67**, 425, author reply 426, doi: 10.1158/0008-5472.can-06-3037 (2007). 10.1158/0008-5472.CAN-06-3037 PMC1941787 17210725

[3] Serafini P, Borrello I, Bronte V (2006). Myeloid suppressor cells in cancer: recruitment, phenotype, properties, and mechanisms of immune suppression. Seminars in cancer biology.

[4] Mocellin S, Bronte V, Nitti D (2007). Nitric oxide, a double edged sword in cancer biology: searching for therapeutic opportunities. Medicinal research reviews.

[5] Krawczyk CM (2010). Toll-like receptor-induced changes in glycolytic metabolism regulate dendritic cell activation. Blood.

[6] Van den Bossche J, Baardman J, de Winther MP (2015). Metabolic Characterization of Polarized M1 and M2 Bone Marrow-derived Macrophages Using Real-time Extracellular Flux Analysis. Journal of visualized experiments: JoVE.

[7] Hammami I, Chen J, Bronte V, DeCrescenzo G, Jolicoeur M (2012). L-glutamine is a key parameter in the immunosuppression phenomenon. Biochemical and biophysical research communications.

[8] Hammami I (2012). Immunosuppressive activity enhances central carbon metabolism and bioenergetics in myeloid-derived suppressor cells *in vitro* models. BMC cell biology.

[9] Robitaille J, Chen J, Jolicoeur M (2015). A Single Dynamic Metabolic Model Can Describe mAb Producing CHO Cell Batch and Fed-Batch Cultures on Different Culture Media. PloS one.

[10] Ghorbaniaghdam A, Henry O, Jolicoeur M (2013). A kinetic-metabolic model based on cell energetic state: study of CHO cell behavior under Na-butyrate stimulation. Bioprocess and biosystems engineering.

[11] Ghorbaniaghdam A, Chen J, Henry O, Jolicoeur M (2014). Analyzing clonal variation of monoclonal antibody-producing CHO cell lines using an in silico metabolomic platform. PloS one.

[12] Hensley CT (2016). Metabolic Heterogeneity in Human Lung Tumors. Cell.

[13] Gentric, G., Mieulet, V. & Mechta-Grigoriou, F. Heterogeneity in Cancer Metabolism: New Concepts in an Old Field. *Antioxid Redox Signal*. Epub ahead of print (2016). 10.1089/ars.2016.6750 PMC5359687 27228792

[14] Marigo I (2010). Tumor-induced tolerance and immune suppression depend on the C/EBPbeta transcription factor. Immunity.

[15] Kimball E, Rabinowitz JD (2006). Identifying decomposition products in extracts of cellular metabolites. Analytical biochemistry.

[16] Cloutier M, Perrier M, Jolicoeur M (2007). Dynamic flux cartography of hairy roots primary metabolism. Phytochemistry.

[17] Cloutier M (2009). Kinetic metabolic modelling for the control of plant cells cytoplasmic phosphate. Journal of theoretical biology.

[18] Ghorbaniaghdam A, Henry O, Jolicoeur M (2014). An in-silico study of the regulation of CHO cells glycolysis. Journal of theoretical biology.

[19] Hundal HS, Rennie MJ, Watt PW (1989). Characteristics of acidic, basic and neutral amino acid transport in the perfused rat hindlimb. The Journal of Physiology.

[20] Ahn WS, Antoniewicz MR (2011). Metabolic flux analysis of CHO cells at growth and non-growth phases using isotopic tracers and mass spectrometry. Metabolic engineering.

[21] Viale A, Corti D, Draetta GF (2015). Tumors and Mitochondrial Respiration: A Neglected Connection. Cancer research.

[22] Desideri E, Vegliante R, Ciriolo MR (2015). Mitochondrial dysfunctions in cancer: genetic defects and oncogenic signaling impinging on TCA cycle activity. Cancer letters.

[23] Lehninger, A. *Biochemistry*. (Worth, 1977).

[24] Koivunen P (2007). Inhibition of hypoxia-inducible factor (HIF) hydroxylases by citric acid cycle intermediates: possible links between cell metabolism and stabilization of HIF. The Journal of biological chemistry.

[25] Corzo CA (2010). HIF-1alpha regulates function and differentiation of myeloid-derived suppressor cells in the tumor microenvironment. The Journal of experimental medicine.

[26] Benito, A., Diaz-Moralli, S., Coy, J., Centelles, J. & Cascante, M. In *Tumor Cell Metabolism: Pathways, Regulation and Biology* (eds Sybille Mazurek & Maria Shoshan) 143–163 (2015).

[27] Murphy C, Newsholme P (1998). Importance of glutamine metabolism in murine macrophages and human monocytes to L-arginine biosynthesis and rates of nitrite or urea production. Clinical science (London, England: 1979).

[28] Draghiciu O, Lubbers J, Nijman HW, Daemen T (2015). Myeloid derived suppressor cells-An overview of combat strategies to increase immunotherapy efficacy. Oncoimmunology.

[29] Germain RN, Meier-Schellersheim M, Nita-Lazar A, Fraser IDC (2011). Systems Biology in Immunology – A Computational Modeling Perspective. Annu Rev Immunol..

