# Supplementary material for: A Dynamic Metabolic Flux Analysis of Myeloid-Derived Suppressor Cells Confirms Immunosuppression-Related Metabolic Plasticity
Source: Sci Rep. 2017 Aug 29;7:9850. doi: 10.1038/s41598-017-10464-1 (PMC5575287; doi:10.1038/s41598-017-10464-1)
Supplement: Supplementary file 1 — Supplementary information [file 41598_2017_10464_MOESM1_ESM.pdf]

# A DYNAMIC METABOLIC FLUX ANALYSIS OF MYELOID-DERIVED SUPPRESSOR CELLS CONFIRMS IMMUNOSUPPRESSION-RELATED METABOLIC PLASTICITY

Guillaume Goffaux, Iness Hammami, Mario Jolicoeur*

Research Laboratory in Applied Metabolic Engineering, Department of Chemical Engineering, École Polytechnique de Montréal, Montréal, Quebéc, Canada.

## Supplementary Information

* Corresponding author

E-mail : mario.jolicoeur@polymtl.ca

# Supplementary Information I : Details on model development

In this section, the detailed procedure of building the model is described. Several steps are commented from the model structure based on mass balances to the parameter identification process through the sensitivity analysis.

## Model structure

The model structure can be described by a set of differential equations resulting from the mass balance principle. There are three categories of variables representing concentrations: the $M_{int}$ intracellular metabolites $M_{int} \in \Re^{M_{int}}$ which are measured in $mmol$ by $10^6$ $cells$, the $M_{ext}$ extracellular $M_{ext} \in \Re^{M_{ext}}$ entering the cells (expressed in $mM$) and finally the cells density $X \in \Re$ ($10^6 cells\ / mL$).

The differential equations are then represented by:

$$\frac{d}{dt} M_{int}(t) = S \cdot v(t) - \mu(t) \cdot M_{int}(t) - \mu_{growth\_M_{int}} \quad (1)$$

$$\frac{d}{dt} M_{ext}(t) = S \cdot v(t) \cdot X(t) \cdot 1000 \text{ mL / L} \quad (2)$$

$$\frac{d}{dt} X(t) = \mu X(t) \quad (3)$$

In these expressions, $\mu \in \Re$ is the specific growth rate ($h^{-1}$). Eq. (1) contains two terms:

- A reaction term with $S \in \Re^{M_{int} \times M_r}$ the stoichiometric matrix and $v \in \Re^{M_r}$ the vector of $M_r$ reaction rates.
- A dilution term: $\mu(t)\ M_{int}(t)$.
- And a term accounting for the consumption rate ($\mu_{growth\_M_{int}}$) of the $M_{int}$ metabolite being integrated into the macromolecules composing the cell mass; and which is therefore no more available for the reactions.

In addition, in Eq. (2), $M_{ext} \in \Re^{M_{ext} \times M_r}$ is the stoichiometric matrix linking the extracellular metabolites to the vector of reaction rates.

The reaction rates are modelled by Michaelis-Menten type equations:

$$v_j(t) = \bar{v_j} \prod_{k \in M_j} \frac{M_k}{M_k + K_{j,k}} \qquad j \in 1, \ldots, M_r \quad (4)$$

$M_k$ is the k$^{th}$ element of the vector $\boldsymbol{M}$ containing all the component concentrations:
$\boldsymbol{M^T} = \left[M_{int}^T, M_{ext}^T, X\right] \in \Re^{M_x}$. The superscript symbol $T$ means the transpose operator.

In Eq. (4), the subscript j indicates the j$^{th}$ element of the vector $v$ containing the $M_r$ reaction rates. $\bar{v_j}$ is the maximum flux rate of the j$^{th}$ kinetic rate. $K_{j,k}$ is the affinity constant related to the metabolite $M_k$ in the j$^{th}$ kinetic rate. $M_j$ refers to the set of indexes related to the metabolites used to characterize the j$^{th}$ kinetic rate.

In order to clarify the notations, the example of the first reaction rate $\mathrm{V_{HK}}$ is selected in Table 2. It has two affinity constants $\mathrm{K_{1,EGLC}}$ and $\mathrm{K_{1,ATP}}$ related to two variables ($EGLC$ and $ATP$). The index of each variable could be for instance 1 and 40 in the vector $\boldsymbol{M}$. Consequently, the notation becomes:

- $v_1 = \mathrm{V_{HK}}$ and $\bar{v_1} = \mathrm{V_{max,HK}}$ for the reaction rate and its maximum value
- $x_1 = EGLC$ , $M_{40} = ATP$ for the component concentrations
- $n_1 = \{1{,}40\}$ the selected indexes
- $K_{1,1} = \mathrm{K_{1,EGLC}}$ and $K_{1,40} = \mathrm{K_{1,ATP}}$ for the affinity constants.

Parameter identification

Once the model structure is defined, the parameter identification is performed with the available measurements. Basically, the identification is performed by minimizing the sum of squares of the errors between the measurements and the predicted values given by the model. The parameter identification can be formulated as below:

$$\min_{\theta_{sub}} \sum_{k=1}^{n_y} J_k(\theta) \tag{5}$$

$$J_k(\theta) = \sum_{i=1}^{n_t} \left(y_{meas,k}(t_i) - y_k(\theta, t_i)\right)^2$$

In Eq. (5), $n_y$ is the number of measured variables. In general, the set of measured variables $\left(\boldsymbol{y}^T = \left[y_1, \dots, y_{n_y}\right]\right)$ is a subset of the vector **x** of the component concentrations. The measurement at time $t_i$ of the $k^{th}$ measured variable is noted $y_{meas,k}(t_i)$. $y_k(\theta, t_i)$ is the $k^{th}$ measured variable at time $t_i$ deduced from the model (integration of the differential equations) with $\theta$ as parameter vector. $\theta_{sub}$ is a subset of the whole parameter vector $\theta$. $\theta$ contains all the maximum flux rates and the affinity constants.

$$\theta^T = [\bar{v}^T, K^T].$$

$$\bar{v}^T = [\bar{v}_1, \dots, \bar{v}_{44}]$$
$$K^T = \left[K_{1,1}, K_{1,40}, \dots, K_{44,2}, K_{44,5}, K_{44,17}\right] \tag{6}$$

In Eq. (6) the last reaction rate is the growth rate: $\left(v_{44} = \mathrm{V_{growth}}\right)$ and the variables have the following notation: $x_2 = G6P$, $x_5 = R5P$ , $x_{17} = EGLC$.

In general, as the number of parameters is substantial, a subset of parameters is used, fixing the others at the previous given or estimated values.

Sensibility analysis

A study of the parameters sensibility is performed to determine the most sensitive parameters. In order to estimate the parameter sensitivity, a cost function $J$ is defined based on the weighted distance

(noted $e$) between the initial model prediction (using $\theta_0$ as a set of parameters) and another model prediction using another set of parameters (noted $\theta$).

$$J(\theta) = \sqrt{\frac{1}{n_x \times n_t} \sum_{j=1}^{n_x} \sum_{k=1}^{n_t} e_j^2(\theta, t_k)} \text{ avec } e_j(\theta, t_k) = \frac{1}{w_j} \left( x_j(\theta_0, t_k) - x_j(\theta, t_k) \right) \quad (7)$$

In Eq. (6), $w_j$ is a weight related to the j$^{th}$ variable. The objective of the chosen weights is to scale the values of metabolites evolving with time to have similar quantities. For instance the weight $w_j$ can be the value at the initial instant $t_0$ of the variable $x_j$ (w_j=x_j (θ_0,t_0)) or its mean value.

The sensitivity analysis allows selecting the most sensitive parameters which can be a first choice in the parameter estimation process.

Strategy of parameter estimation

The strategy of the parameter estimation is schematized by the following diagram. The main strategy is to perform a sensitivity analysis to determine a hierarchy in the parameters. The maximum flux rates are ranked according to their sensitivity. Knowing the hierarchy, parameter subsets are defined containing a small number of parameters to optimize (for instance 8 maximum). The strategy proposed by Rizzi et al. (1997) enabled proceeding by steps minimizing the number of parameters at each step.

The choice of the parameters can be determined according to the sensitivity analysis and/or using some knowledge of the metabolic structure. For instance, four sensitive parameters in the set $\overline{\nu}$ are $V_{HK}$, $V_{ATPase}$, $V_{leak}$ and $V_{PFK}$. According to Table 2, they become $\nu_1$, $\nu_{42}$, $\nu_{40}$ and $\nu_6$ respectively. Based on this information, several subsets could be chosen according to the structure of the metabolic network. In this example, $V_{HK}$ and $V_{PFK}$ are in the glycolysis pathways and can be gathered along with their most sensitive affinity constants. Similarly, all the parameters related to $V_{leak}$ and $V_{ATPase}$ could be part of another parameter subset.

Then, when the subsets are selected, an iterative procedure takes place testing all subsets consecutively. For each of them, the optimized subset replaced the original one in the parameter vector $\theta$ after the optimization, in order to be used for the next iteration.

**Reference**

Rizzi M, Baltes M, Theobald U, Reuss M. 1997. In vivo analysis of metabolic dynamics in Saccharomyces cerevisiae: II. Mathematical model. Biotechnol Bioeng 55(4)

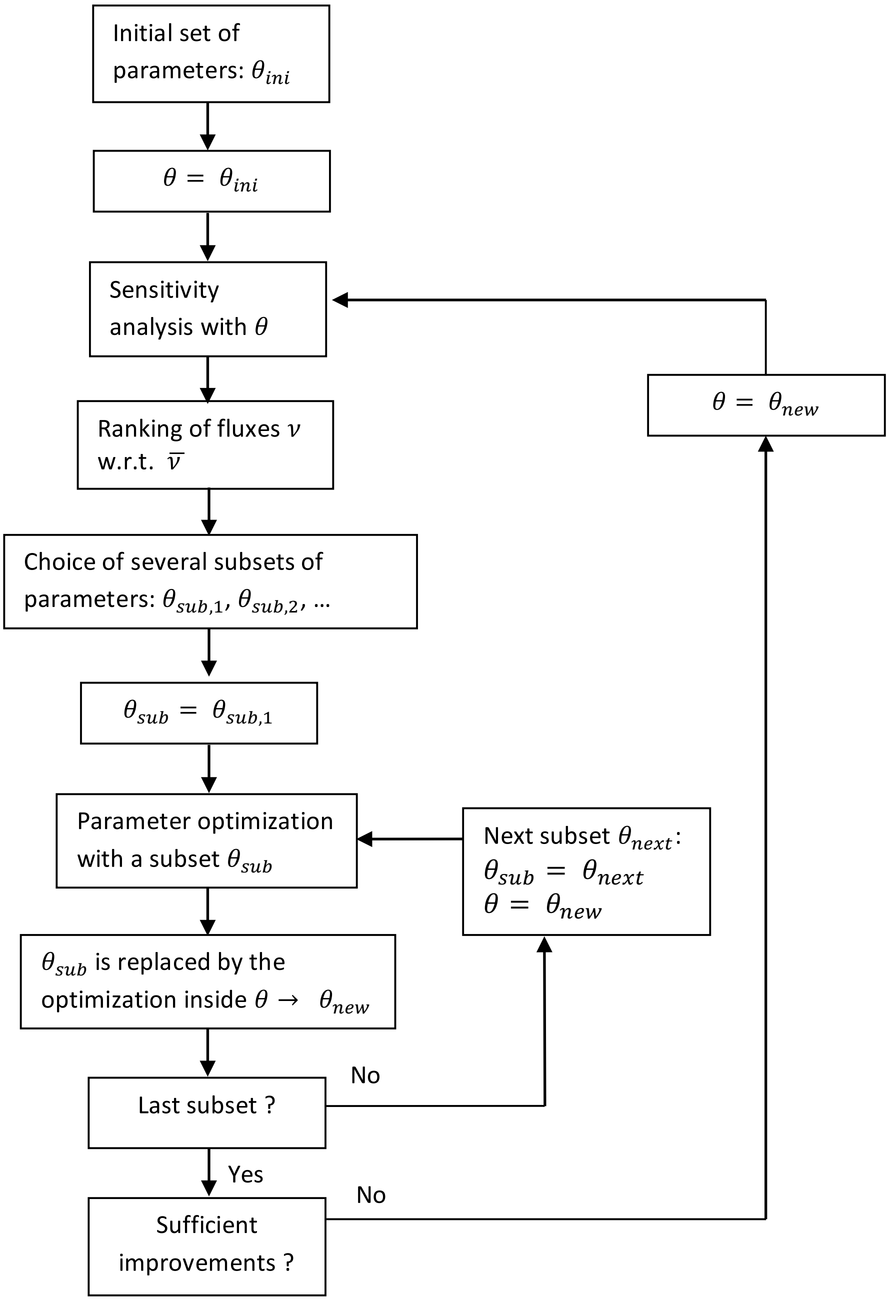


**Figure S1.** Algorithm model structure calibration and parameters value estimation

**Supplementary Information II : Model simulations with no experimental data**

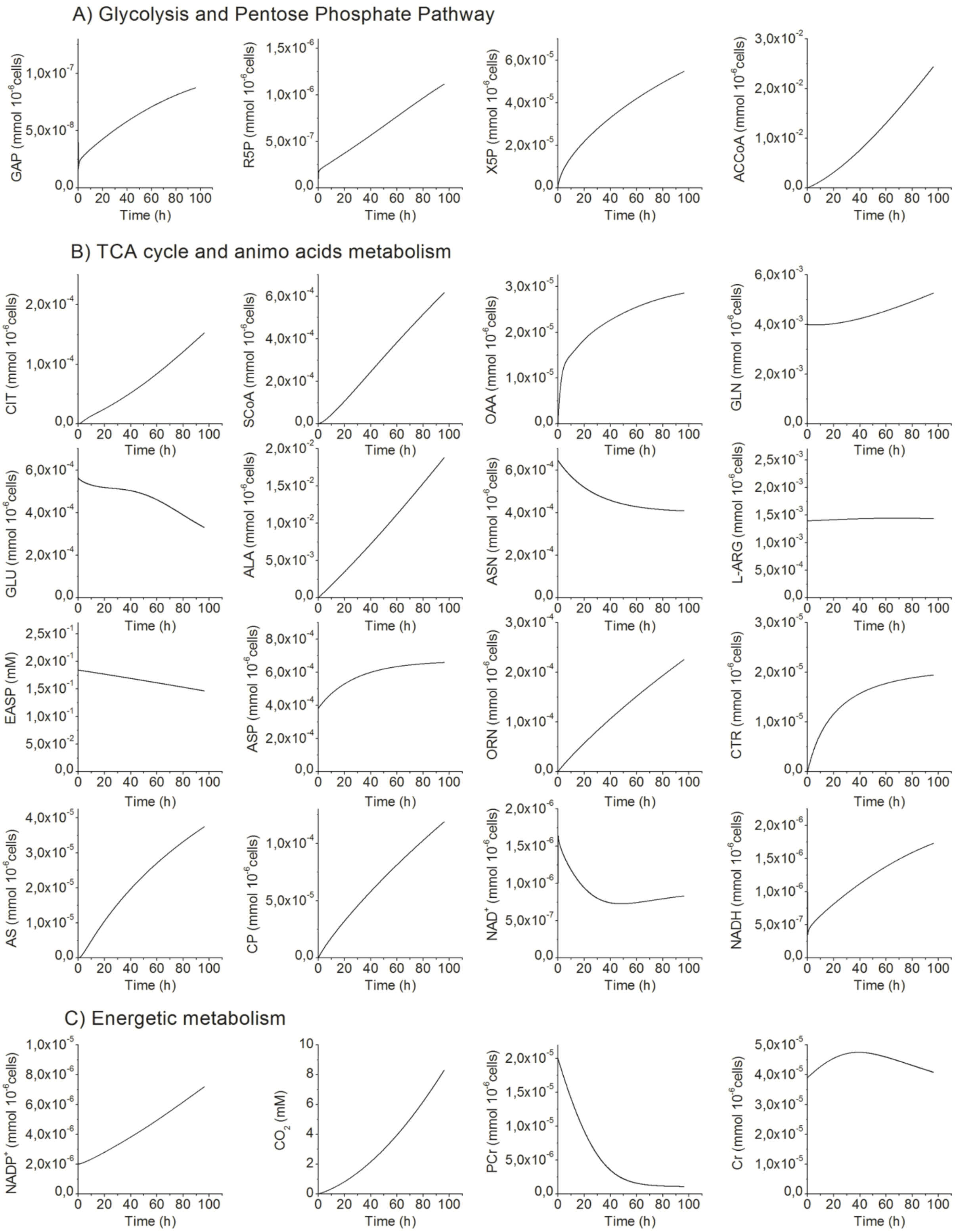


**Figure S2.** Model simulations of metabolites with no experimental data
